# Supplementary material for: Dormant spores sense amino acids through the B subunits of their germination receptors
Source: Nat Commun. 2021 Nov 25;12:6842. doi: 10.1038/s41467-021-27235-2 (PMC8617281; doi:10.1038/s41467-021-27235-2)
Supplement: Supplementary file 1 — Supplementary Information [file 41467_2021_27235_MOESM1_ESM.pdf]

Dormant spores sense amino acids through the B subunits of their germination receptors

Lior Artzi, Assaf Alon, Kelly P. Brock, Anna G. Green, Amy Tam, Fernando H. Ramírez-Guadiana, Debora Marks, Andrew Kruse, David Z. Rudner

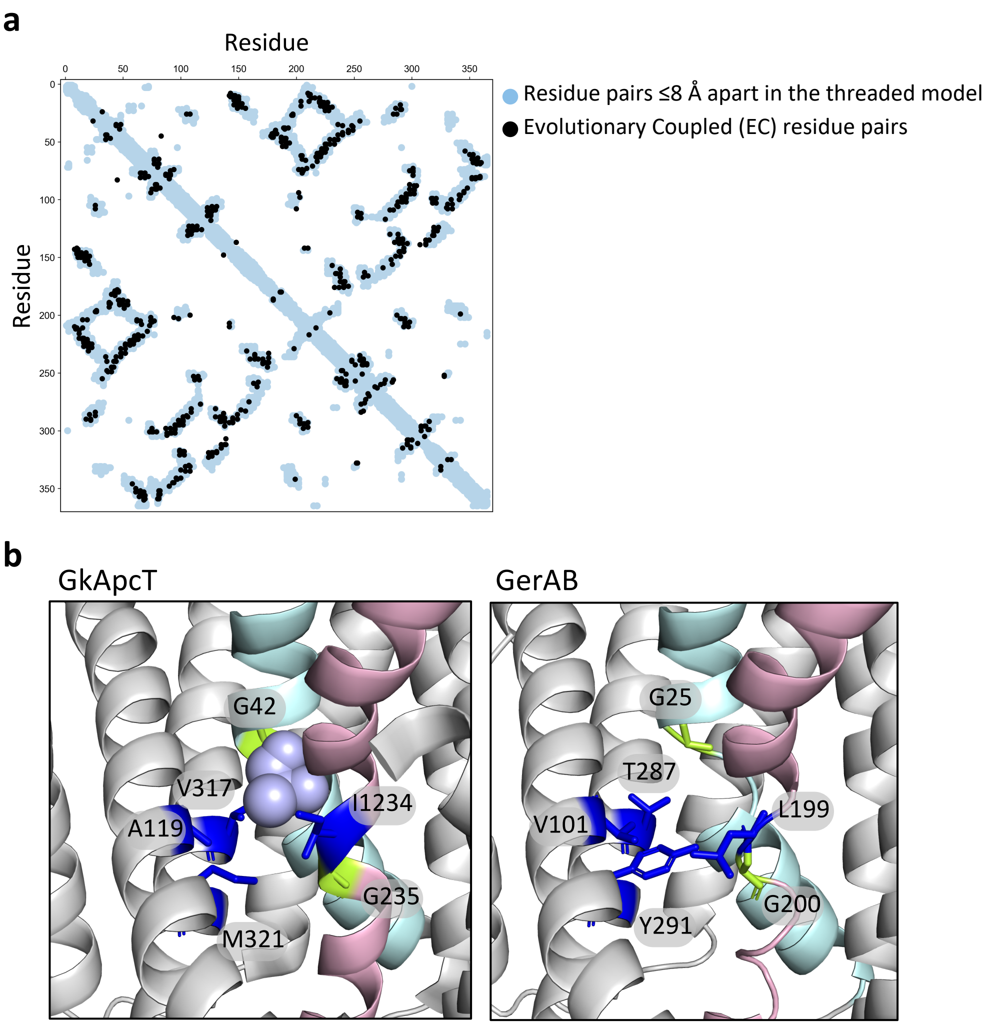

Supplementary Figure 1. GerAB resembles the L-alanine transporter GkApcT. **a**, Interaction matrix comparing evolutionary coupled (EC) residue pairs in GerAB (black circles) with residue pairs that are ≤8 Å apart in the GerAB model (light blue circles) derived from the GkApcT structure. 85% of the EC pairs were within 8 Å of each other in the structural model. **b**, Comparison of the L-alanine binding pocket in GkApcT and the predicted pocket in GerAB. TM segments 1 (cyan) and 6 (pink) are highlighted. The glycines in these TM segments that generate kinks in the helices are shown in green. Residues predicted to line the L-alanine binding pocket are indicated in dark blue. L-alanine is shown in light purple. Side chains of the indicated amino acids are shown for GkApcT and GerAB but should be interpreted cautiously in the case of the GerAB homology model.

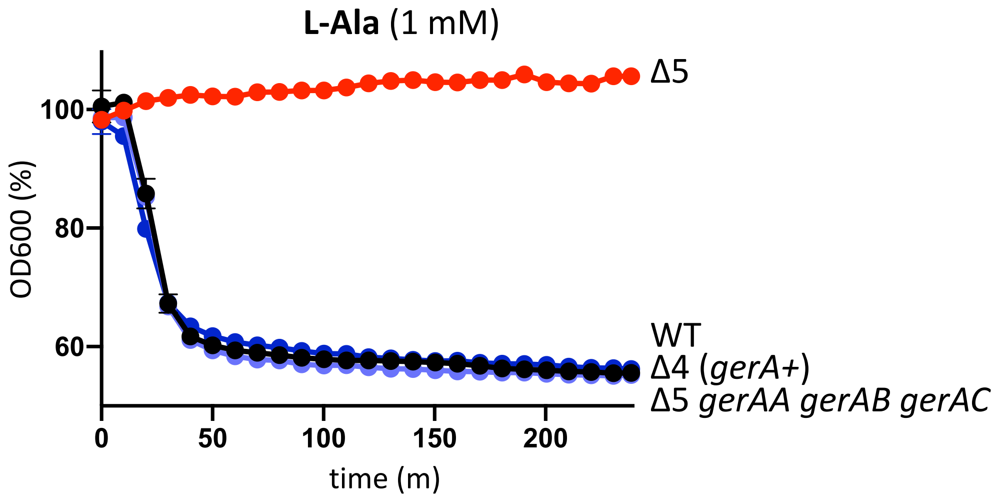

Supplementary Figure 2. Expression of *gerAA*, *gerAB*, and *gerAC* at separate chromosomal loci supports L-alanine germination. Spore germination in response to 1 mM L-alanine was assessed by the percent reduction of initial OD600 over 240 min. Spores lacking all five putative germinant receptor loci (Δ5) do not respond to L-alanine. Spores with an intact *gerA* operon (Δ4 *gerA+*) or the three *gerA* genes expressed at separate chromosomal loci (Δ5 *gerAA gerAB gerAC*) but lacking the other putative receptors germinate with kinetics similar to wild-type (WT). Representative data from one of two biological replicates with two technical replicates are shown. Error bars indicate +/- SD of two technical replicates.

a

	% spore viability
gerAB(WT)	100
Δ*gerAB*	0.18
gerAB(**Y291S**)	0.12

b

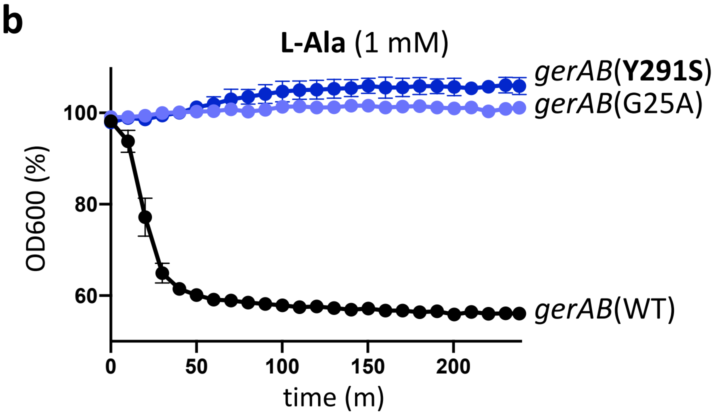

c

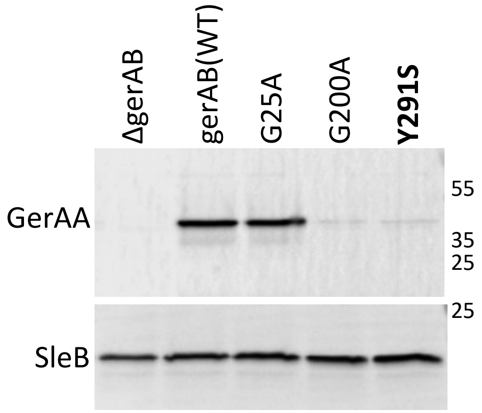

Supplementary Figure 3. GerAA fails to accumulate in spores expressing *gerAB*(Y291S). **a**, Spore viability of the indicated strains following heat treatment (80˚C for 20 min) and colony formation on LB agar plates. **b**, Spore germination in response to 1 mM L-alanine as assessed by the percent reduction in initial OD600. Data from one of two biological replicates each with two technical replicates are shown. Error bars indicate +/- SD of two technical replicates. **c**, Impact of GerAB variants on the stability of GerAA in dormant spores. Immunoblot analysis of spore lysates of the indicated strains using anti-GerAA antibodies. SleB was used to control for loading. Molecular weight markers in Kd are indicated on the right. Representative data from one of three biological replicates are shown. Source data are provided as a Source Data file.

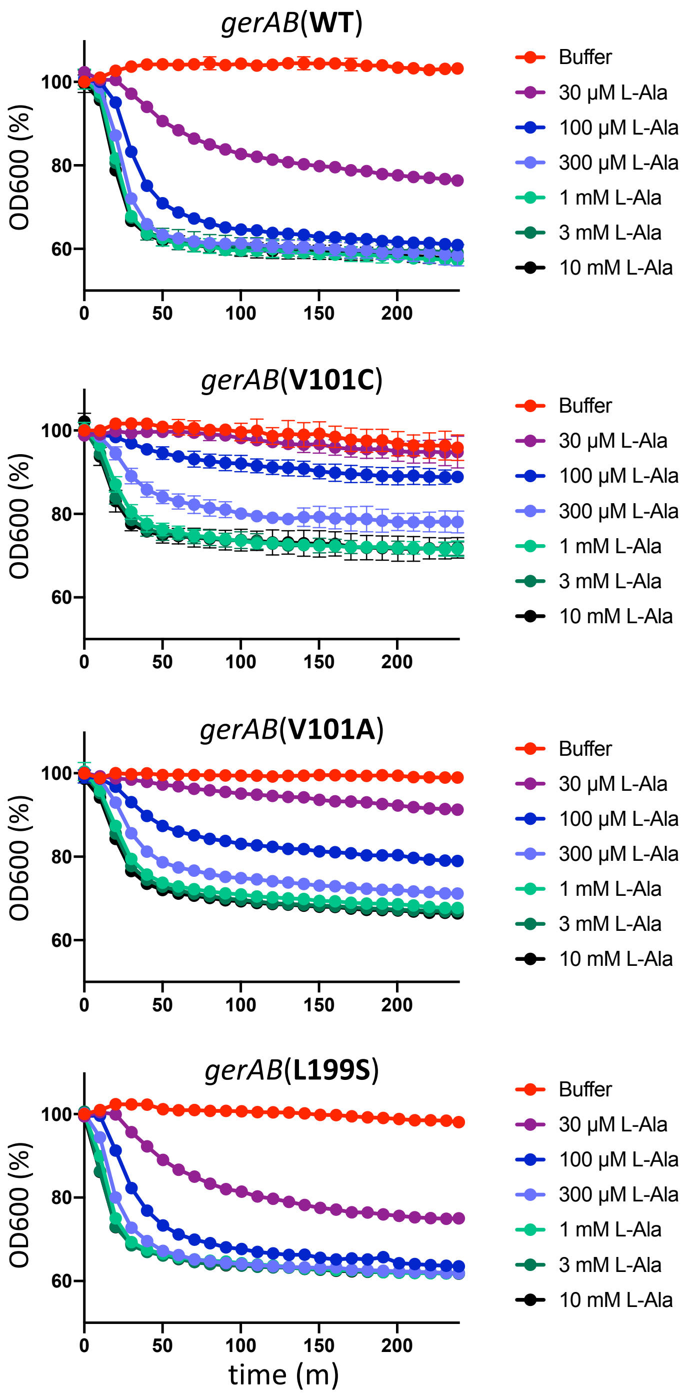

Supplementary Figure 4. Spores harboring GerAB(V101C), GerAB(V101A), or GerAB(L199S) respond to L-alanine with kinetics similar to wild-type. Spore germination of the indicated strains as assayed by the percent reduction in initial OD600 over a 240 min time course. Concentration of L-alanine used to trigger germination varied from 30 µM to 10 mM. The decrease in optical density in the V101 mutants was reproducibly less than wild-type, likely due to spontaneous germination of a subset of the spores during purification. Representative data from one of two biological replicates with two technical replicates are shown. Error bars indicate +/- SD of two technical replicates.

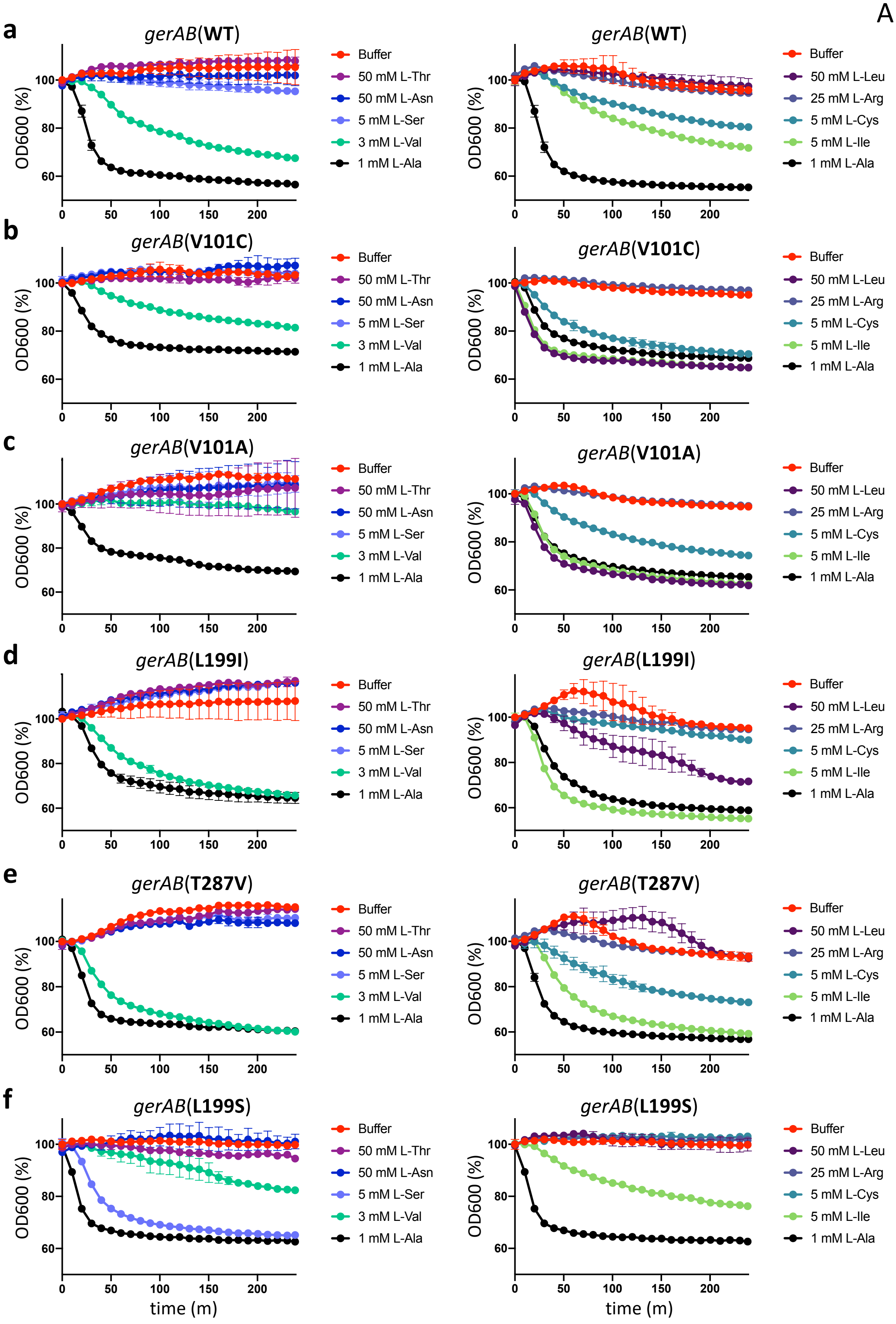

Supplementary Figure 5. Spores harboring GerAB variants with altered germinant specificity respond to most amino acids in a manner similar to wild-type. Spore germination of the indicated strains as assayed by the percent reduction in initial OD600 over a 240 min time course. Concentrations of L-threonine, L-asparagine, L-serine, L-valine, L-alanine, L-leucine, L-arginine, L-cysteine, and L-isoleucine are indicated. The decrease in optical density in the V101 mutants was reproducibly less than wild-type, likely due to spontaneous germination of a subset of the spores during purification. Representative data from one of two biological replicates with two technical replicates are shown. Error bars indicate +/- SD of two technical replicates.

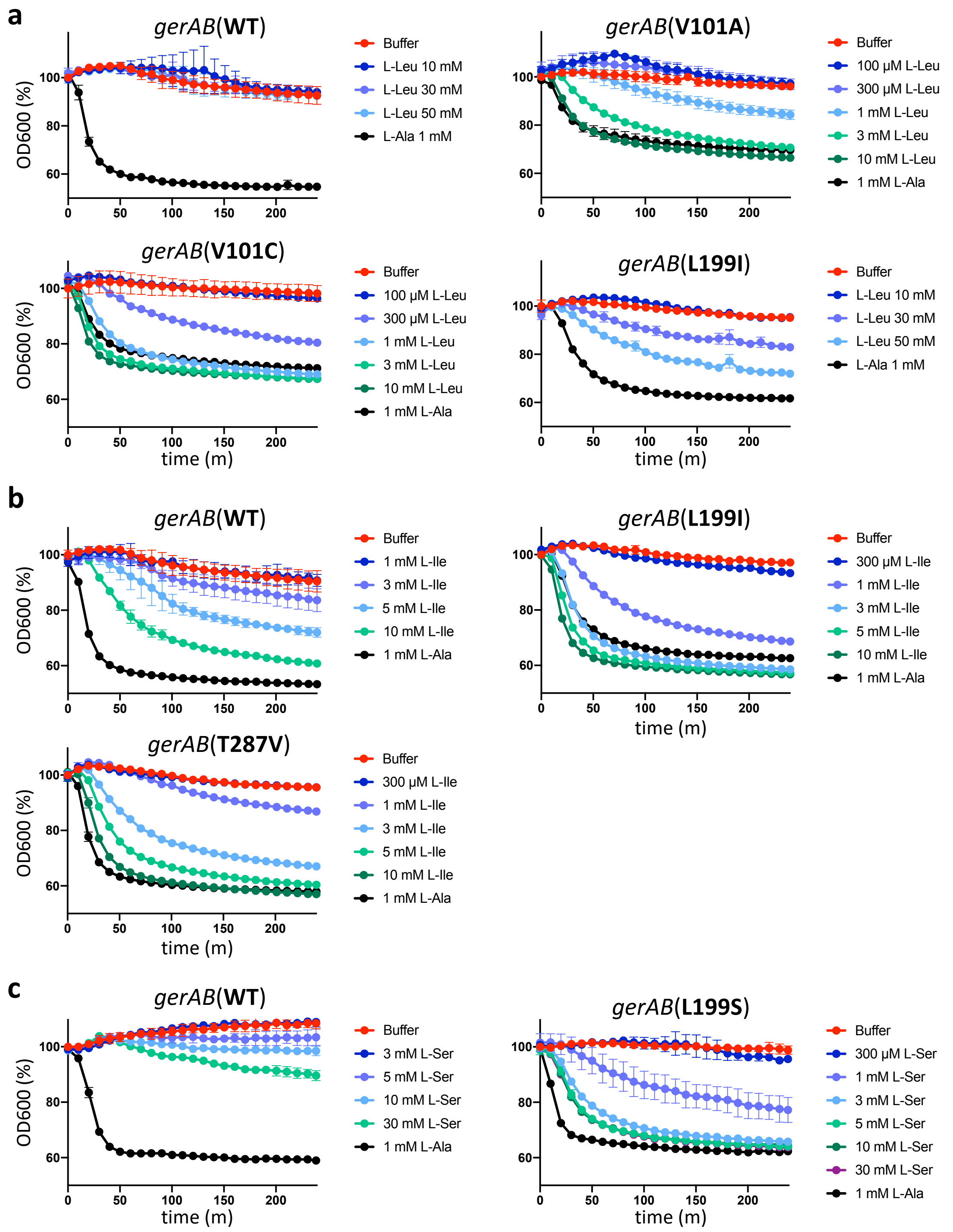

Supplementary Figure 6. Spores harboring GerAB variants with altered germinant specificity respond over a range of amino acid concentrations. **a**, Spore germination of the indicated strains in response to L-leucine. The graphs show the percent reduction in initial OD600 over a 240 min time course in the presence of the indicated concentration of L-leucine. **b**, Spore germination of the indicated strains in response to L-isoleucine. **c**, Spore germination of the indicated strains in response to a range of L-serine concentrations. Representative data from one of two biological replicates with two technical replicates are shown. Error bars indicate +/- SD of two technical replicates.

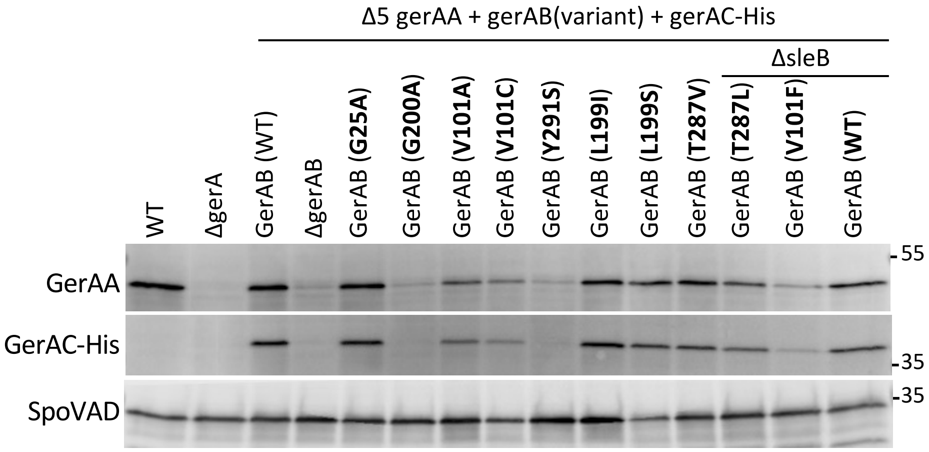

Supplementary Figure 7. Stability of GerAA and GerAC in the GerAB mutants. Immunoblot analysis of spore lysates of the indicated strains using anti-GerAA and anti-His antibodies. The two mutants that prematurely germinate (T287L and V101F) were sporulated in a Δ*sleB* background to enable spore purification. The levels of GerAA and GerAC in the GerAB(V101F) mutant were reduced compared to wild-type suggesting that a low level of the activated GerA receptor is sufficient to trigger germination. SpoVAD was used to control for loading. Molecular weight markers in Kd are indicated on the right. Representative data from one of three biological replicates are shown. Source data are provided as a Source Data file.

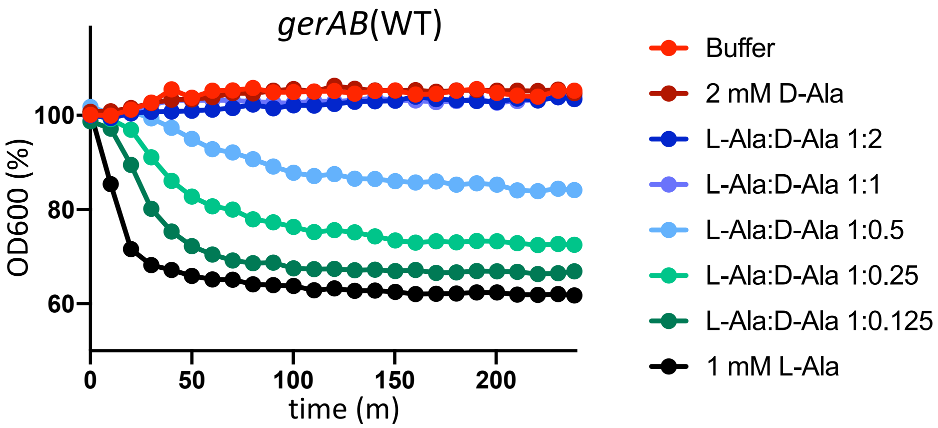

Supplementary Figure 8. D-alanine inhibits germination of spores harboring GerAB(WT) spores in response to L-alanine. Spores were incubated with 1 mM L-alanine with or without D-alanine at the indicated ratios. Representative data from one of two biological replicates are shown.

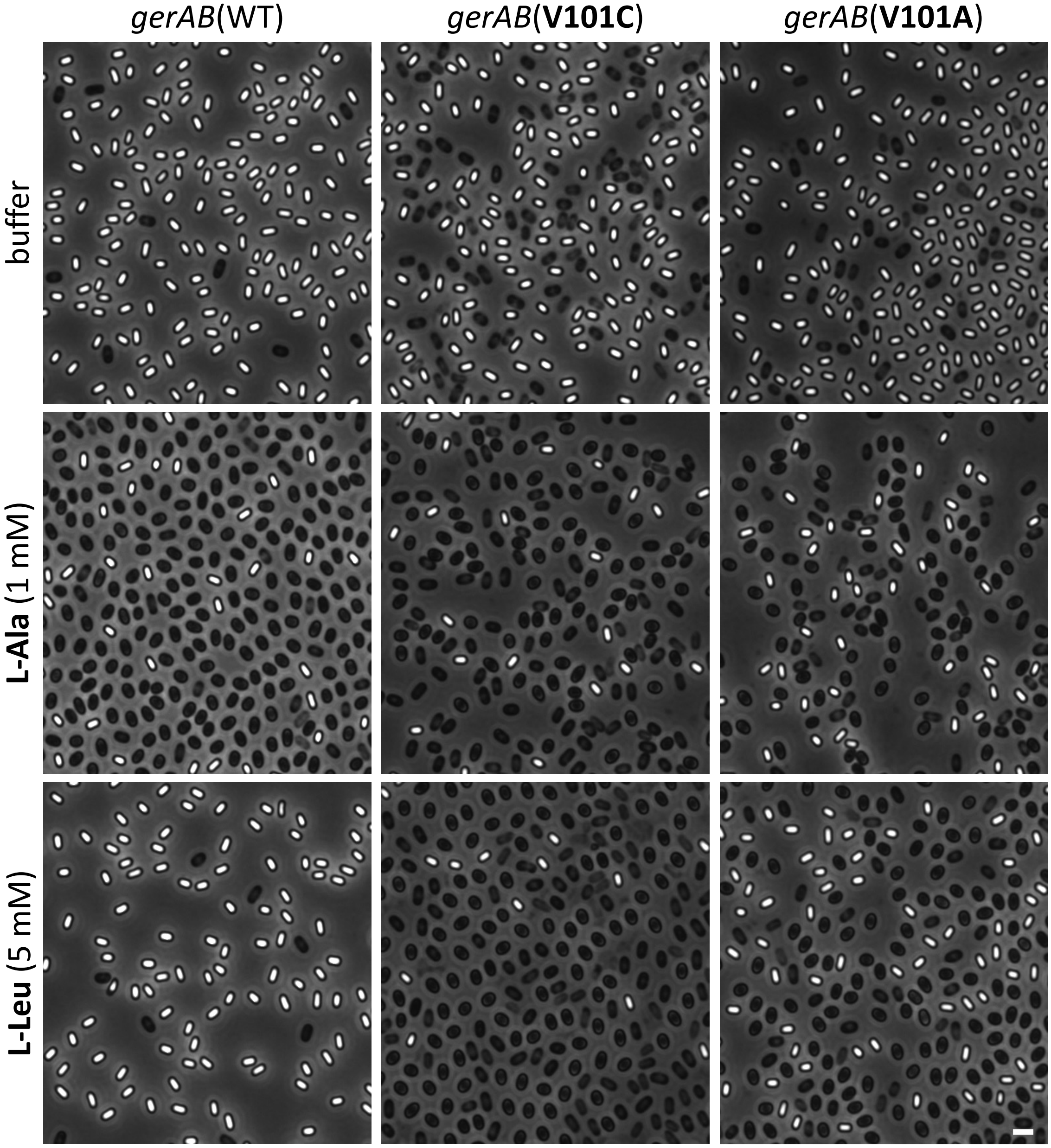

Supplementary Figure 9. Spores harboring GerAB(V101C) and GerAB(V101A) respond to L-leucine. Representative phase-contrast images of wild-type, *gerAB*(V101C), and *gerAB*(V101A) spores after 100 min incubation with buffer, 1 mM L-alanine, or 5 mM L-leucine. Representative data from one of three biological replicates are shown. Scale bar, 2 μm.

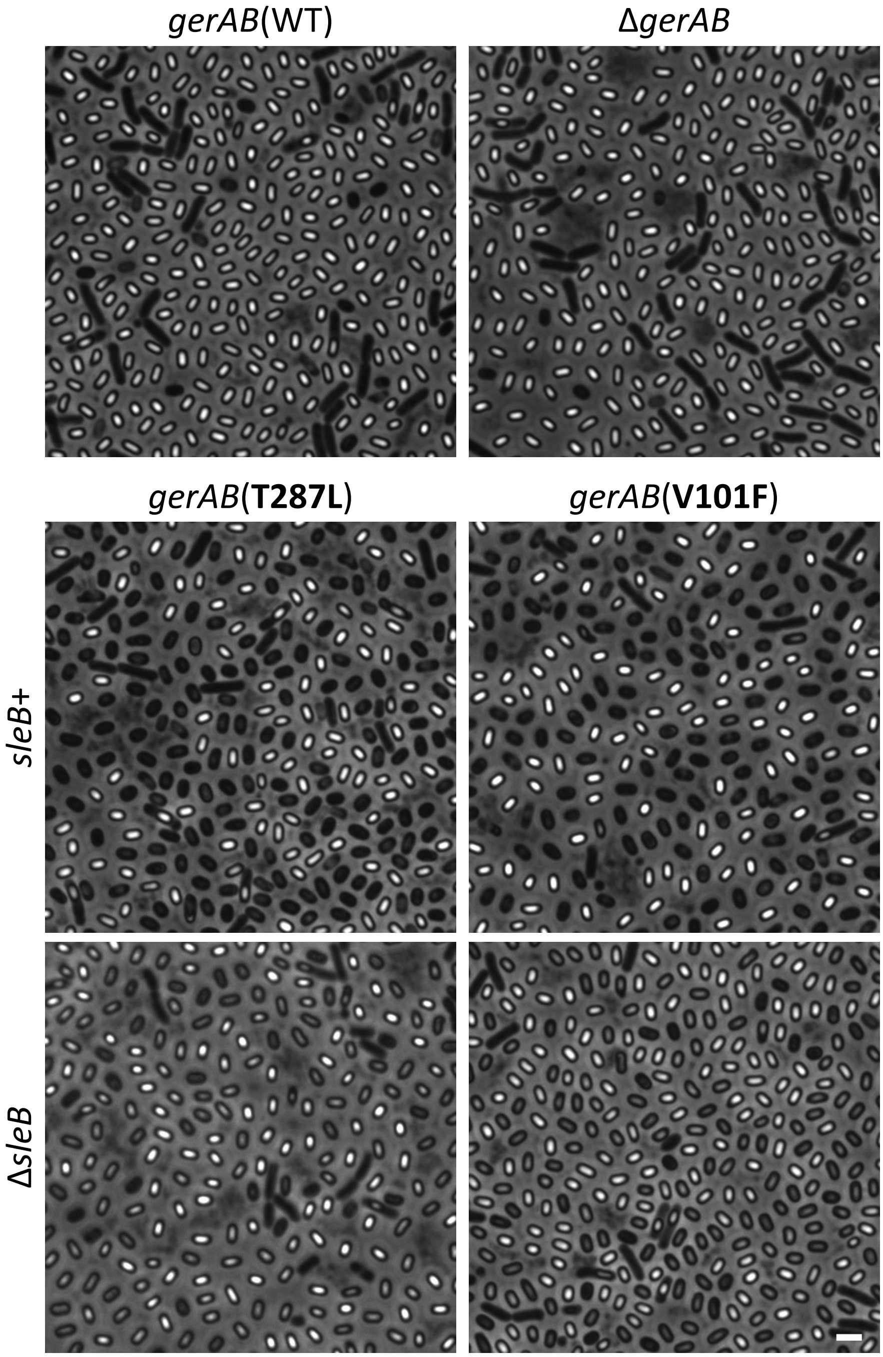

Supplementary Figure 10. Substitutions of bulkier residues in the putative ligand-binding pocket of GerAB cause premature germination. Representative phase-contrast images of indicated strains after 30 h of sporulation. The majority of sporulating cells with wild-type *gerAB* or lacking *gerAB* produce phase-bright spores. Cells expressing *gerAB*(T287L) or *gerAB*(V101F) produce phase-dark spores indicative of premature germination. Deletion of *sleB* encoding the spore cortex hydrolase that is activated during germination suppresses the phase-dark phenotype in the *gerAB* mutants. Representative data from one of three biological replicates are shown. Scale bar, 2 µm.

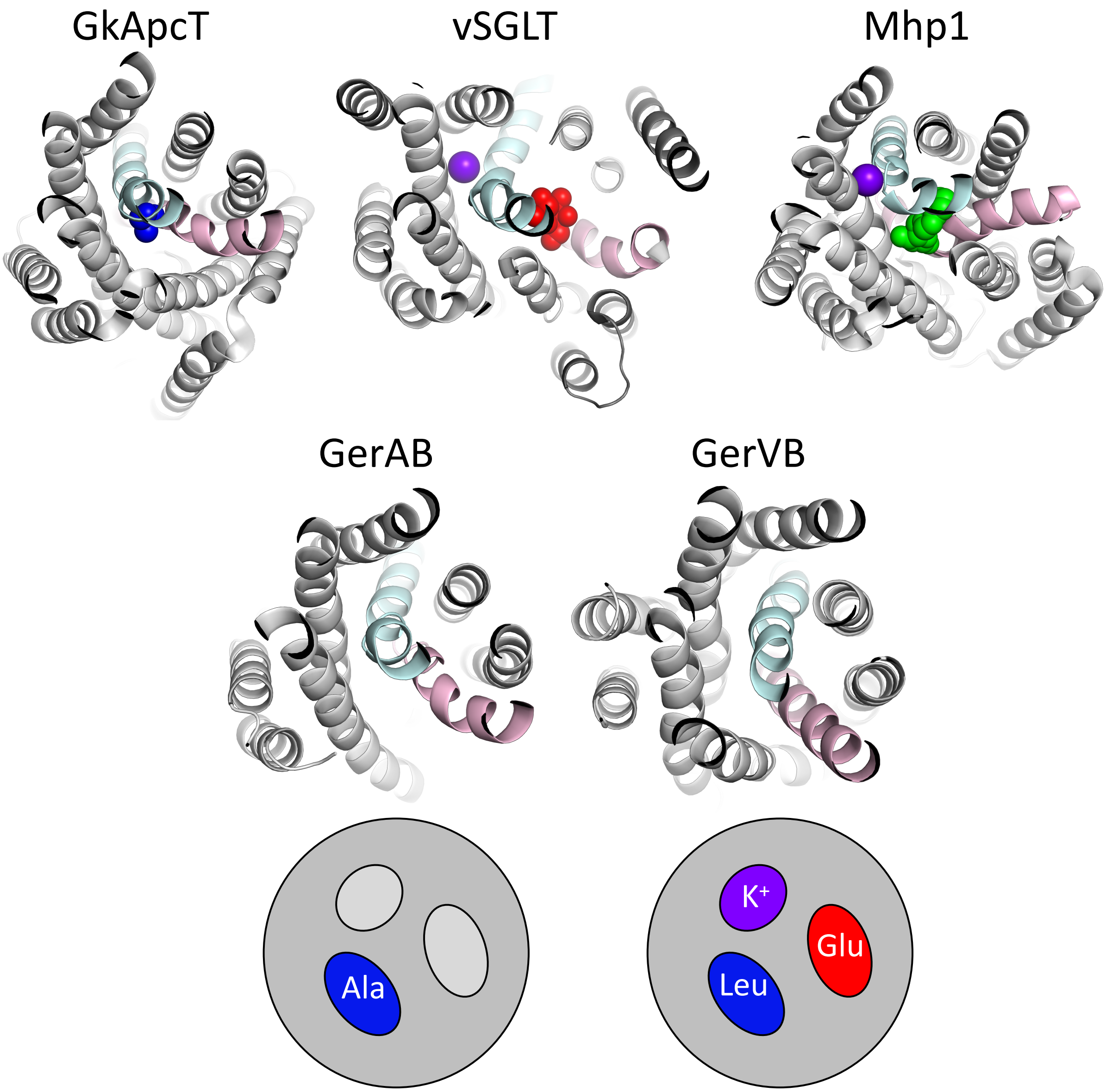

Supplementary Figure 11. APC super-family members have similar structural cores with distinct ligand-binding pockets. Top-down views of GkApcT (PDB:5OQT), vSGLT (PDB:3DH4), Mhp1 (PDB:4D1A), the threaded structure of *B. subtilis* GerAB, and the predicted structure of *B. megaterium* GerVB based on evolutionary co-variation. Extra- and intra-cellular loops have been removed for clarity. TM segments equivalent to TM1 (cyan) and TM6 (pink) in GkApcT are highlighted. L-alanine (dark blue), galactose (red), indolylmethyl-hydantoin (green), and sodium ions (purple) present in the crystal structures are shown. Schematic diagrams below GerAB and GerVB with proposed germinant and co-germinant binding pockets for L-alanine (Ala), L-leucine (Leu), D-glucose (Glu), and monovalent cations (K+).

Supplementary Fig. 12
Artzi et al.

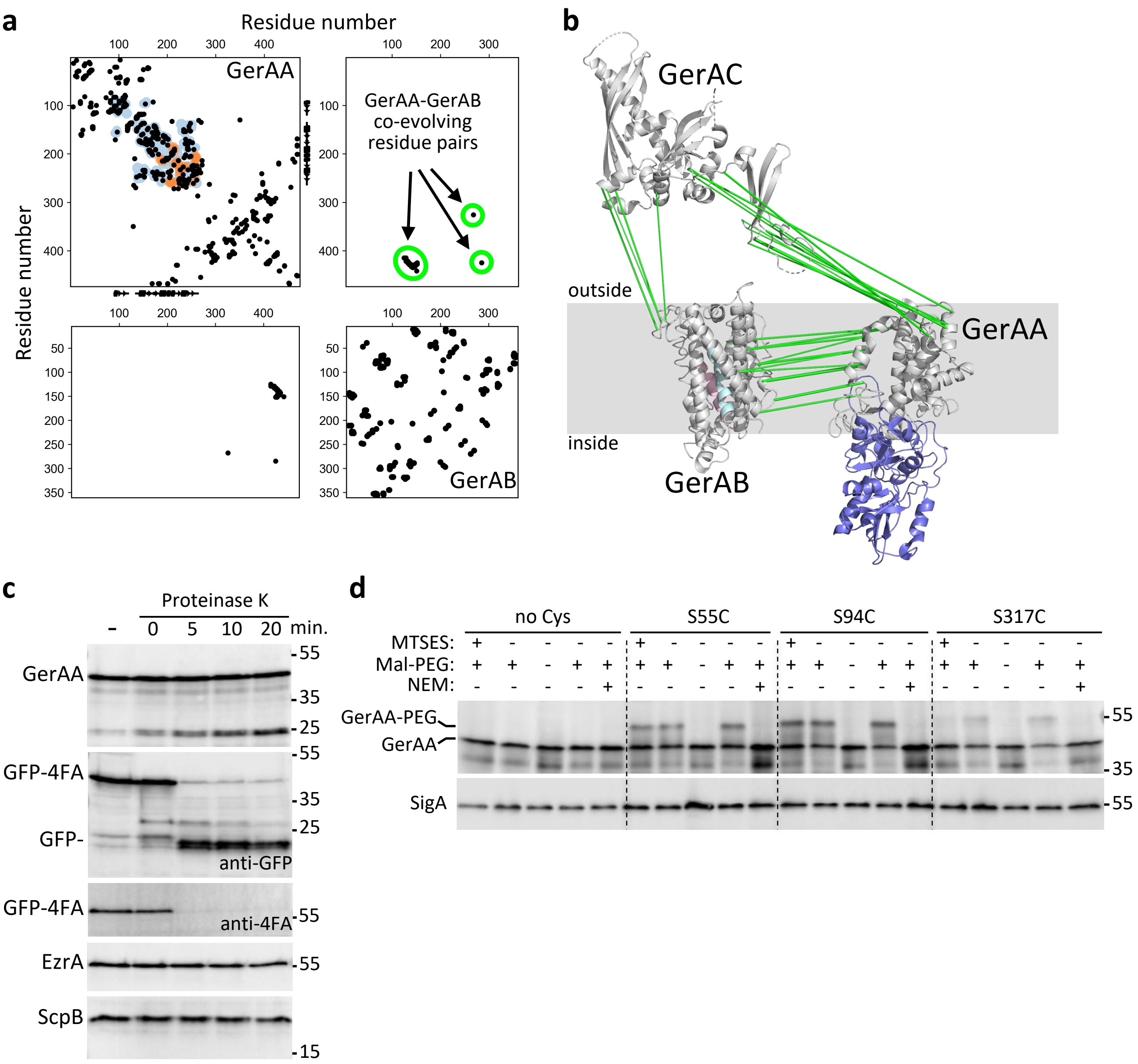

Supplementary Figure 12. The soluble N-terminal domain of GerAA resides in the spore core. **a**, Evolutionarily coupled residue pairs in GerAA, GerAB, and between GerAA and GerAB are plotted as black circles. Residue pairs that are ≤5 Å apart in the GerAA structural model derived from the GerK3A structure (PDB:6O59) are shown as blue circles. Orange circles show residue pairs in adjacent protomers in the crystal. **b**, Models of GerAA and GerAB derived from co-variation analysis. GerAC is based on the crystal structure of GerBC (PDB:3N54). Green lines highlight the evolutionarily coupled residues between subunits in the putative complex. The soluble N-terminal domain of GerAA is highlighted in blue and is predicted to reside in the spore core (inside). **c**, The soluble N-terminal domain of GerAA is inaccessible to Proteinase K. GerAA, GerAB and GerAC were co-expressed in vegetatively growing *B. subtilis* cells. Protoplasts were treated with Proteinase K for the indicated times and lysates resolved by SDS-PAGE. Immunoblot analysis indicates that the majority of GerAA remains full-length during Proteinase K treatment. A small amount of proteolysis occurs generating ~25 Kda cleavage product that is protected from further proteolysis. Since the anti-GerAA antibodies were raised against the soluble N-terminal domain, these data indicate that this domain is located in the cytoplasm. The 25 KDa cleavage product likely results from Proteinase K digestion of a small extracellular loop. The extracellular domain of the sporulation integral membrane protein GFP-SpoIVFA (4FA), expressed under xylose control is efficiently degraded releasing the intracellular GFP-fusion. The membrane protein EzrA that lacks an extracellular domain and the soluble protein ScpB were inaccessible to the protease. The anti-4FA antibodies were raised against the extracytoplasmic soluble C-terminal domain. Representative data are from one of three biological replicates. **d**, Substituted cysteine accessibility assay (SCAM) indicates that that N-terminal domain of GerAA is intracellular. GerAA(C100S) (no Cys) and the indicated cysteine substitutions were co-expressed with GerAB and GerAC in vegetatively growing cells. Protoplasts were incubated with (+) or without (-) the membrane impermeant Cys crosslinker MTSES, or with (+) or without (-) the membrane permeant Cys crosslinker NEM. The protoplasts were lysed and proteins denatured in the presence (+) or absence (-) of Mal-PEG and GerAA was analyzed by immunoblot. S55C and S94C in the soluble N-terminus were inaccessible to MTSES, while S317C, predicted to reside in an extracellular loop, was partially accessible to MTSES and partially blocked Mal-PEG labeling. All Cys residues were blocked in the presence of NEM and were not further labeled by Mal-PEG. Representative data are from one of three biological replicates. All four GerAA variants were functional (Supplementary **Table 1**). For more information about protease accessibility and SCAM assays see Supplementary Methods. Source data are provided as a Source Data file.

Supplementary Table 1

strain	% spore viability
WT	100
Δ4 (gerA+)	91.7
Δ5	0.16
Δ5 gerAA/gerAB/gerAC	97.0
***gerAB* variants**	
Δ5 gerAA/AB(**WT**)/AC	100
Δ5	0.18
Δ5 gerAA/AB(**G25A**)/AC	5.0
Δ5 gerAA/AB(**G200A**)/AC	0.27
Δ5 gerAA/AB(**L199I**)/AC	83.3
Δ5 gerAA/AB(**L199S**)/AC	40.8
Δ5 gerAA/AB(**V101A**)/AC	79.2
Δ5 gerAA/AB(**V101C**)/AC	79.8
Δ5 gerAA/AB(**T287V**)/AC	99.5
Δ5 gerAA/AB(**Y291A**)/AC	0.28
Δ5 gerAA/AB(**Y291S**)/AC	0.12
Δ5 gerAA/AB(**T287L**)/AC	2.9
Δ5 gerAA/AB(**V101F**)/AC	12.8
***gerAA* variants**	
Δ5	0.29
Δ5 gerAA(**WT**)/AB/AC	100
Δ5 gerAA(**C100S**)/AB/AC	>100
Δ5 gerAA(**S55C, C100S**)/AB/AC	>100
Δ5 gerAA(**S94C, C100S**)/AB/AC	>100
Δ5 gerAA(**C100S, S317C**)/AB/AC	32.5

Supplementary Table 1. Spore viability of the mutants in this study. Cells were sporulated by nutrient exhaustion in DS medium (DSM) at 37˚ C for 48 h. Spore viability was determined by comparing heat resistant (80˚ C for 20 min) colony forming units (CFUs) of the mutants to wild-type. Sporulated cultures of *B. subtilis* wild-type and strains containing *gerA* as the sole germinant receptor (native or complemented) have similar survival percentages following heat treatment. Spore viability data of the indicated strains with mutations in *gerAB* and *gerAA* are presented. Representative data are from one of three biological replicates.

Supplementary Table 2

GerAB mutant	Phenotypes
V101L	Spore germination in response to 15 mM L-leucine and slightly less responsive to L-cysteine. Spore viability at T30 based on heat-kill assay: 84.15%
T287S	Spore germination response was similar to WT except the mutant did not respond to 5 mM L-Ileucine. Spore viability at T30 based on heat-kill assay: 78%
V101S	Spore germination response was reduced to all amino acids tested (including L-Alanine). Spore viability at T30 based on heat-kill assay: 23.75%.
V101T	Spore germination in response to 50 mM L-leucine. Spore viability at T30 based on heat-kill assay: 57.5%
Y291A	Spore germination response was impaired with all amino acids tested (including L-alanine). Spore viability at T30 based on heat-kill assay: 0.3%.
L199V	Some phase-dark spores at T30 indicative of premature germination. Spore viability at T30 based on heat-kill assay: 50%.
L199A	Some phase-dark spores at T30 indicative of premature germination. Spore viability at T30 based on heat-kill assay: 66%.
I196A	Spore germination in response to L-alanine similar to WT. Non-responsive to L-Ser, L-Ile, L-Val, L-Cys. Spore viability at T30 based on heat-kill assay: 93.58%.
G25V	GerAA subunit unstable based on immunoblot as reported previously by A. Moir. Spore viability at T30 based on heat-kill assay: 0.65%.

Supplementary Table 2. Summary of GerAB mutants tested but not analyzed in detail. This table describes the GerAB mutants that were generated and underwent initial characterization but were not rigorously analyzed.

Supplementary Table 3 – List of strains used in this study

All strains were derived from *Bacillus subtilis* 168 (*trpC2*)

Strain	Genotype	Source	Figure(s)
BDR2413	Wild-type *Bacillus subtilis* 168 (*trpC2*)	Zeigler *et al.* 2008[1]	S2, S7, Table S1
BAM841	*ΔgerBB::lox72 ΔgerKB::lox72 ΔyfkT::lox72 ΔyndE::lox72* **(Δ4 *gerA+*)**	Ramírez-Guadiana *et al.*, 2017[2]	S2, Table S1
bLA201	*ΔgerBB::lox72 ΔgerKB::lox72 ΔyfkT::lox72 ΔyndE::lox72 ΔgerA::lox72* **(Δ5)**	This work	2, 3, 4, S2, S3, S7, S10, Table S1
bLA204	*ΔgerBB, ΔgerKB, ΔyfkT, ΔyndE, ΔgerA, lacA::spec*	This work	
bLA207	*ΔgerBB, ΔgerKB, ΔyfkT, ΔyndE, ΔgerA, lacA::spec, ycgO::kan*	This work	
bLA210	*ΔgerBB, ΔgerKB, ΔyfkT, ΔyndE, ΔgerA, lacA::spec, ycgO::kan, yhdG::cat*	This work	
bLA212	*ΔgerBB, ΔgerKB, ΔyfkT, ΔyndE, ΔgerA, lacA::PgerA-gerAC(erm), ycgO::kan, yhdG::cat*	This work	
bLA215	*ΔgerBB, ΔgerKB, ΔyfkT, ΔyndE, ΔgerA, lacA::PgerA-gerAC(erm), ycgO::PgerA-gerAB(spec), yhdG::cat*		
bLA216	*ΔgerBB, ΔgerKB, ΔyfkT, ΔyndE, ΔgerA, lacA::PgerA-gerAC(erm), ycgO::kan, yhdG::PgerA-gerAA(tet)* **(Δ5 gerAA/AC)**	This work	
bLA219	*ΔgerBB, ΔgerKB, ΔyfkT, ΔyndE, ΔgerA, lacA::PgerA-gerAC(erm), ycgO::PgerA-gerAB(spec), yhdG::PgerA-gerAA(tet)* **(Δ5 gerAA/AB/AC**)	This work	2, 3, 4, S2, S3, S4, S5, S6, S8, S9, S10, Table S1
bLA286	*ΔgerBB, ΔgerKB, ΔyfkT, ΔyndE, ΔgerA, lacA::PgerA-gerAC(erm), ycgO::PgerA-gerAB*(**G25A**)*(spec), yhdG::PgerA-gerAA(tet)* **(Δ5 gerAA/AB(G25A)/AC**)	This work	2, S3, Table S1
bLA287	*ΔgerBB, ΔgerKB, ΔyfkT, ΔyndE, ΔgerA, lacA::PgerA-gerAC(erm), ycgO::PgerA-gerAB*(**V101A**)*(spec), yhdG::PgerA-gerAA(tet)* **(Δ5 gerAA/AB(V101A)/AC**)	This work	3, S4, S5, S6, S9, Table S1
bLA289	*ΔgerBB, ΔgerKB, ΔyfkT, ΔyndE, ΔgerA, lacA::PgerA-gerAC(erm), ycgO::PgerA-gerAB*(**V101C**)*(spec), yhdG::PgerA-gerAA(tet)* **(Δ5 gerAA/AB(V101C)/AC**)	This work	3, S4, S5, S6, S9, Table S1
bLA291	*ΔgerBB, ΔgerKB, ΔyfkT, ΔyndE, ΔgerA, lacA::PgerA-gerAC(erm), ycgO::PgerA-gerAB*(**G200A**)*(spec), yhdG::PgerA-gerAA(tet)* **(Δ5 gerAA/AB(G200A)/AC**)	This work	2, S3, Table S1
bLA292	*ΔgerBB, ΔgerKB, ΔyfkT, ΔyndE, ΔgerA, lacA::PgerA-gerAC(erm), ycgO::PgerA-gerAB*(**Y291S**)*(spec), yhdG::PgerA-gerAA(tet)* **(Δ5 gerAA/AB(Y291S)/AC**)	This work	S3, Table S1
bLA295	*ΔgerBB, ΔgerKB, ΔyfkT, ΔyndE, ΔgerA, lacA::PgerA-gerAC(erm), ycgO::PgerA-gerAB*(**L199I**)*(spec), yhdG::PgerA-gerAA(tet)* **(Δ5 gerAA/AB(L199I)/AC)**	This work	S5, S6, Table S1
bLA296	*ΔgerBB, ΔgerKB, ΔyfkT, ΔyndE, ΔgerA, lacA::PgerA-gerAC(erm), ycgO::PgerA-gerAB*(**L199S**)*(spec), yhdG::PgerA-gerAA(tet)* **(Δ5 gerAA/AB(L199S)/AC**)	This work	3, S4, S5, S6, Table S1
bLA303	*ΔgerBB, ΔgerKB, ΔyfkT, ΔyndE, ΔgerA, lacA::PgerA-gerAC(erm), ycgO::PgerA-gerAB*(**T287V**)*(spec), yhdG::PgerA-gerAA(tet)* **(Δ5 gerAA/AB(T287V)/AC**)	This work	S5, S6, Table S1
bLA304	*ΔgerBB, ΔgerKB, ΔyfkT, ΔyndE, ΔgerA, lacA::PgerA-gerAC(erm), ycgO::PgerA-gerAB*(**T287L**)*(spec), yhdG::PgerA-gerAA(tet)* **(Δ5 gerAA/AB(T287L)/AC**)	This work	4, S10, Table S1

bLA308	*ΔgerBB, ΔgerKB, ΔyfkT, ΔyndE, ΔgerA, lacA::PgerA-gerAC(erm), ycgO::PgerA-gerAB*(**V101F**)*(spec), yhdG::PgerA-gerAA(tet)*<br>**(Δ5 gerAA/AB(V101F)/AC**)	This work	4, S10, Table S1
bLA315	*ΔgerBB, ΔgerKB, ΔyfkT, ΔyndE, ΔgerA, lacA::PgerA-gerAC(erm), ycgO::PgerA-gerAB*(**T287L**)*(spec), yhdG::PgerA-gerAA(tet), sleB::kan*<br>**(Δ5 gerAA/AB(T287L)/AC ΔsleB**)	This work	4, S10
bLA316	*ΔgerBB, ΔgerKB, ΔyfkT, ΔyndE, ΔgerA, lacA::PgerA-gerAC(erm), ycgO::PgerA-gerAB*[**V101F**]*-lox-spec, yhdG::PgerA-gerAA-lox-tet, sleB::kan*<br>**(Δ5 gerAA/AB[V101F]/AC ΔsleB**)	This work	4, S10
bLA321	*ΔgerBB, ΔgerKB, ΔyfkT, ΔyndE, ΔgerA, lacA::PgerA-gerAC-His(phleo), ycgO::PgerA-gerAB(spec), yhdG::PgerA-gerAA(tet)*<br>**(Δ5 gerAA/AB/AC-His)**	This work	2, S7
bLA322	*ΔgerBB, ΔgerKB, ΔyfkT, ΔyndE, ΔgerA, lacA::PgerA-gerAC-His(phleo), ycgO::kan, yhdG::PgerA-gerAA(tet)*<br>**(Δ5 gerAA/AC-His)**	This work	2, S7
bLA323	*ΔgerBB, ΔgerKB, ΔyfkT, ΔyndE, ΔgerA, lacA::PgerA-gerAC-His(phleo), ycgO::PgerA-gerAB*(**G25A**)*(spec), yhdG::PgerA-gerAA(tet)*<br>**(Δ5 gerAA/AB(G25A)/AC-His)**	This work	2, S7
bLA324	*ΔgerBB, ΔgerKB, ΔyfkT, ΔyndE, ΔgerA, lacA::PgerA-gerAC-His(phleo), ycgO::PgerA-gerAB*(**G200A**)*(spec), yhdG::PgerA-gerAA(tet)*<br>**(Δ5 gerAA/AB(G200A)/AC-His)**	This work	2, S7
bLA328	*ΔgerBB, ΔgerKB, ΔyfkT, ΔyndE, ΔgerA, lacA::PgerA-gerAC(erm), ycgO::PgerA-gerAB(spec), yhdG::PgerA-gerAA*(**C100S**)*(tet)*<br>**(Δ5 gerAA(C100S)/AB/AC)**	This work	Table S1
bLA335	*ΔgerBB, ΔgerKB, ΔyfkT, ΔyndE, ΔgerA, lacA::PgerA-gerAC(erm), ycgO::PgerA-gerAB(spec), yhdG::PgerA-gerAA*(**S55C, C100S**)*(tet)*<br>**(Δ5 gerAA(S55C, C100S)/AB/AC)**	This work	Table S1
bLA336	*ΔgerBB, ΔgerKB, ΔyfkT, ΔyndE, ΔgerA, lacA::PgerA-gerAC(erm), ycgO::PgerA-gerAB(spec), yhdG::PgerA-gerAA*(**S94C, C100S**)*(tet)*<br>**(Δ5 gerAA(S94C, C100S)/AB/AC)**	This work	Table S1
bLA338	*ΔgerBB, ΔgerKB, ΔyfkT, ΔyndE, ΔgerA, lacA::PgerA-gerAC(erm), ycgO::PgerA-gerAB(spec), yhdG::PgerA-gerAA*(**C100S, S317C**)*(tet)*<br>**(Δ5 gerAA(C100S, S317C)/AB/AC)**	This work	Table S1
bLA358	*ΔgerBB, ΔgerKB, ΔyfkT, ΔyndE, ΔgerA, lacA::Pveg-gerAC-His(phleo), ycgO::Pveg-gerAB(spec), yhdG::Pveg-gerAA*(**C100S**)*(tet)*	This work	S12
bLA359	*ΔgerBB, ΔgerKB, ΔyfkT, ΔyndE, ΔgerA, lacA::Pveg-gerAC-His(phleo), ycgO::Pveg-gerAB(spec), yhdG::Pveg-gerAA*(**S55C, C100S**)*(tet)*	This work	S12
bLA360	*ΔgerBB, ΔgerKB, ΔyfkT, ΔyndE, ΔgerA, lacA::Pveg-gerAC-His(phleo), ycgO::Pveg-gerAB(spec), yhdG::Pveg-gerAA*(**S94C, C100S**)*(tet)*	This work	S12
bLA362	*ΔgerBB, ΔgerKB, ΔyfkT, ΔyndE, ΔgerA, lacA::Pveg-gerAC-His(phleo), ycgO::Pveg-gerAB(spec), yhdG::Pveg-gerAA*(**C100S, S317C**)*(tet)*	This work	S12
bLA363	*ΔgerBB, ΔgerKB, ΔyfkT, ΔyndE, ΔgerA, lacA::Pveg-gerAC-His(phleo), ycgO::Pveg-gerAB(spec), yhdG::Pveg-gerAA*(**C100S**)*(tet), amyE::PxylA-gfp-spoIVFA(cat)*	This work	S12

bLA368	*ΔgerBB, ΔgerKB, ΔyfkT, ΔyndE, ΔgerA, lacA::PgerA-gerAC-His(phleo), ycgO::PgerA-gerAB*(**V101A**)*(spec), yhdG::PgerA-gerAA(tet)*<br>**(Δ5 gerAA/AB(V101A)/AC-His**)	This work	S7
bLA369	*ΔgerBB, ΔgerKB, ΔyfkT, ΔyndE, ΔgerA, lacA::PgerA-gerAC-His(phleo), ycgO::PgerA-gerAB*(**V101C**)*(spec), yhdG::PgerA-gerAA(tet)*<br>**(Δ5 gerAA/AB(V101C)/AC-His**)	This work	S7
bLA370	*ΔgerBB, ΔgerKB, ΔyfkT, ΔyndE, ΔgerA, lacA::PgerA-gerAC-His(phleo), ycgO::PgerA-gerAB*(**Y291S**)*(spec), yhdG::PgerA-gerAA(tet)*<br>**(Δ5 gerAA/AB(Y291S)/AC-His**)	This work	S7
bLA371	*ΔgerBB, ΔgerKB, ΔyfkT, ΔyndE, ΔgerA, lacA::PgerA-gerAC-His(phleo), ycgO::PgerA-gerAB*(**L199I**)*(spec), yhdG::PgerA-gerAA(tet)*<br>**(Δ5 gerAA/AB(L199I)/AC-His)**	This work	S7
bLA372	*ΔgerBB, ΔgerKB, ΔyfkT, ΔyndE, ΔgerA, lacA::PgerA-gerAC-His(phleo), ycgO::PgerA-gerAB*(**L199S**)*(spec), yhdG::PgerA-gerAA(tet)*<br>**(Δ5 gerAA/AB(L199S)/AC-His**)	This work	S7
bLA373	*ΔgerBB, ΔgerKB, ΔyfkT, ΔyndE, ΔgerA, lacA::PgerA-gerAC-His(phleo), ycgO::PgerA-gerAB*(**T287V**)*(spec), yhdG::PgerA-gerAA(tet)*<br>**(Δ5 gerAA/AB(T287V)/AC-His**)	This work	S7
bLA374	*ΔgerBB, ΔgerKB, ΔyfkT, ΔyndE, ΔgerA, lacA::PgerA-gerAC-His(phleo), ycgO::PgerA-gerAB*(**T287L**)*(spec), yhdG::PgerA-gerAA(tet), sleB::kan*<br>**(Δ5 gerAA/AB(T287L)/AC-His ΔsleB**)	This work	S7
bLA375	*ΔgerBB, ΔgerKB, ΔyfkT, ΔyndE, ΔgerA, lacA::PgerA-gerAC-His(phleo), ycgO::PgerA-gerAB*[**V101F**]*-lox-spec, yhdG::PgerA-gerAA-lox-tet, sleB::kan*<br>**(Δ5 gerAA/AB(V101F)/AC-His ΔsleB**)	This work	S7
bLA376	*ΔgerBB, ΔgerKB, ΔyfkT, ΔyndE, ΔgerA, lacA::PgerA-gerAC-His(phleo), ycgO::PgerA-gerAB(spec), yhdG::PgerA-gerAA(tet), sleB::kan*<br>**(Δ5 gerAA/AB/AC-His ΔsleB)**	This work	S7

Supplementary Table 4. Plasmids constructed in this work

**Plasmid**	**Genotype**	**Source**
pLA010	*ycgO::PgerA (spec) (amp)*	This work
pLA013	*ycgO::PgerA-RBSgerAB-gerAB (spec) (amp)*	This work
pLA023	*lacA::PgerA-RBSgerAC-gerAC (erm) (amp)*	This work
pLA039	*yhdG::PgerA-RBSgerAB-gerAA (tet) (amp)*	This work
pLA099	*ycgO::PgerA-RBSgerAB-gerAB*(G25A) *(spec) (amp)*	This work
pLA100	*ycgO::PgerA-RBSgerAB-gerAB*(V101A) *(spec) (amp)*	This work
pLA102	*ycgO::PgerA-RBSgerAB-gerAB*(V101C) *(spec) (amp)*	This work
pLA104	*ycgO::PgerA-RBSgerAB-gerAB*(G200A) *(spec) (amp)*	This work
pLA105	*ycgO::PgerA-RBSgerAB-gerAB*(Y291S) *(spec) (amp)*	This work
pLA120	*ycgO::PgerA-RBSgerAB-gerAB*(L199I) *(spec) (amp)*	This work
pLA121	*ycgO::PgerA-RBSgerAB-gerAB*(L199S) *(spec) (amp)*	This work
pLA124	*ycgO::PgerA-RBSgerAB-gerAB*(T287V) *(spec) (amp)*	This work
pLA125	*ycgO::PgerA-RBSgerAB-gerAB*(T287L) *(spec) (amp)*	This work
pLA129	*ycgO::PgerA-RBSgerAB-gerAB*(V101F) *(spec) (amp)*	This work
pLA131	*lacA::PgerA-RBSgerAC-gerAC-His (phleo) (amp)*	This work
pLA137	*yhdG::PgerA-RBSgerAB-gerAA*(C100S)*(tet) (amp)*	This work
pLA138	*yhdG::PgerA-RBSgerAB-gerAA*(S55C, C100S)*(tet) (amp)*	This work
pLA139	*yhdG::PgerA-RBSgerAB-gerAA*(S94C, C100S)*(tet) (amp)*	This work
pLA141	*yhdG::PgerA-RBSgerAB-gerAA*(C100S, S317C)*(tet) (amp)*	This work
pLA150	*yhdG::Pveg-RBSgerAB-gerAA*(C100S) *(tet) (amp)*	This work
pLA151	*yhdG::Pveg-RBSgerAB-gerAA*(S55C, C100S) *(tet) (amp)*	This work
pLA152	*yhdG::Pveg-RBSgerAB-gerAA*(S94C, C100S) *(tet) (amp)*	This work
pLA154	*yhdG::Pveg-RBSgerAB-gerAA*(C100S, S317C) *(tet) (amp)*	This work
pLA155	*ycgO::Pveg-RBSgerAB-gerAB (spec) (amp)*	This work
pLA156	*lacA::Pveg-RBSgerAC-gerAC-His (phleo) (amp)*	This work
pDR124	*amyE::PxylA-gfp-spoIVFA (cat) (amp)*	Doan *et al*., 2005[3]
pDR244	P_{PA}*-cre-ori(ts)(spec) (amp)*	Meeske *et al*., 2015[4]

Supplementary Table 5. List of oligonucleotide primers used in this study

Primer	Sequence	genes
oJM28	TTCTGCTCCCTCGCTCA	*cat/kan/phleo* cassette (isothermal assembly)
oJM29	CAGGGAGCACTGGTCAAC	*cat/kan/phleo* cassette (isothermal assembly)
oFR5	TGAATGGTTTCTTTATTAGGC	*gerAA* (isothermal assembly)
oFR6	CTGAGCGAGGGAGCAGAACAATGAGGTCACCTCTTATC	*gerA::cat* (isothermal assembly)
oFR7	GTTGACCAGTGCTCCCTGTAGCAGCCGCCTAATTCAC	*gerA::cat* (isothermal assembly)
oFR8	GTTTCGCCTCAGGGTATATG	*gerAC* (isothermal assembly)
oLA041	GCCGAATTCGCTGTTCAAATGATCTAATGAGCTGTTC	*PgerA* 5'
oLA042	CGCACTAGTAAAACCTTAGTAGAGGTTATCTTTTCCTGT	*PgerA* 3'
oLA046	CGCACTAGTAACCAAAAGAGGTGAATAATCCAAATGAGC	PCR of *gerAB* with its native RBS 5'
oLA047	GCGGGATCCTCATTTTGTTGTAATCCTCCTCTTGAGAGC	PCR of *gerAB.* 3'
oLA048	CGCACTAGTGCTCTCAAGAGGAGGATTACAACAAAATG	PCR of *gerAC* with its native RBS 5'
oLA049	CGCGGATCCCTATTTGTTTGCGCCTTTCGTTCC	PCR of *gerAC* 3'
oLA089	CCGCTCGAGGCTGTTCAAATGATCTAATGAGCTGTTC	PCR of *PgerA-gerAC* 5'
oLA103	GGCACTAGTAACCAAAAGAGGTGAATAATCCAATGGAACAAACAGAGTTTAAGGAATATATACACG	PCR of *RBSgerAB-gerAA* 5’
oLA112	GGCGGATCCAGCCGTCAGGCGTATTAGCC	PCR of *gerAA* 3'
oLA197	AAATACAATGCTTGCGGCCGGACTTTTAACA	PCR mutagenesis *gerAB*(G25A)
oLA198	TATTTCCTCGGCGCGGCCAGCTTCGAGAC	PCR mutagenesis *gerAB*(V101A)
oLA200	TATTTCCTCGGCTGCGCCAGCTTCGAGACACG	PCR mutagenesis *gerAB*(V101C)
oLA202	GTTTCCATCTCTTTTTTAGCGATGGAAGTGATGCTGTTTCTTCC	PCR mutagenesis *gerAB*(G200A)
oLA203	CACCACATTTGTCATTAGCGGATACTTTGCCG	PCR mutagenesis *gerAB*(Y291S)
oLA283	CTGAGCGAGGGAGCAGAATGCATCTTTTCAAGCCTCCTACTGC	*sleB*::kan (isothermal assembly)
oLA284	GATCATCCAAACTATATGGGCGTACC	*ypdA* (isothermal assembly-5' of *sleB*)
oLA285	GTTGACCAGTGCTCCCTGTAGCAGATATGAGAAAGCATAAAAAGAGGTGTG	*sleB::kan* (isothermal assembly)
oLA286	TTGGCATTGATATACATTCTGAATGTGTC	*ypeB* (isothermal assembly – 3' of *sleB*)
oLA293	CGTTTCCATCTCTTTTATTGGAATGGAAGTGATGC	PCR mutagenesis gerAB(L199I)
oLA294	CGTCGTTTCCATCTCTTTTAGCGGAATGGAAGTGATGCTG	PCR mutagenesis *gerAB*(L199S)
oLA297	GGATTATCCAGTTTTTCACCGTCTTTGTCATTTACGGATACTTTGCC	PCR mutagenesis *gerAB*(T287V)
oLA298	GATTATCCAGTTTTTCACCCTGTTTGTCATTTACGGATACTTTGCC	PCR mutagenesis gerAB(T287L)
oLA302	CTATTTCCTCGGCTTTGCCAGCTTCGAGACAC	PCR mutagenesis *gerAB*(V101F)
oLA308	ATTGGATCCTCAATGGTGATGGTGATGGTGGCTGCCTTTGTTTGCGCCTTTCGTTCCG	PCR of *gerAC* with C-terminal 6XHis tag
oLA359	CCGGAATTCTTGAAAACCTGCATAGGAGAGCTATG	PCR of Pveg promoter. 5'
oLA360	CCGACTAGTCACTACATTTATTGTACAACACGAGCCC	PCR of Pveg promoter. 3'
oLA365	CCGCTCGAGTTGAAAACCTGCATAGGAGAGCTATG	PCR of Pveg promoter. 5'
oLA323	CGATTCTCAACGGCAATTCAGCTGTGTTTATCAACG	PCR mutagenesis *gerAA*(C100S)
oLA324	AATGACTGATGAGAATAAGGTGTGCGAAGCCATTAAAACTTTGATT	PCR mutagenesis *gerAA*(S55C)
oLA340	GTTGAAACTGCAAAAAAAACGATTGAATGCATTCTCAACGGCAATTCAGCTGTG	PCR mutagenesis *gerAA*(S94C)
oLA327	GCCGTCACGATTTGCGCGAACAGGGAAAACG	PCR mutagenesis *gerAA*(S317C)

Restriction endonuclease recognition sites are underlined.

Supplementary Methods

Strain constructions

bLA201 [Δ*gerBB::lox72,* Δ*gerKB::lox72,* Δ*yfkT::lox72,* Δ*yndE::lox72,* Δ*gerA::lox72*] was generated by transforming *B. subtilis* BAM841 [Δ*gerBB::lox72,* Δ*gerKB::lox72,* Δ*yfk::lox72,* Δ*yndE::lox72*] with the isothermal assembly product *gerA::cat* generated by three PCR products. The three PCR products were amplified with: (1) oFR5 (*fumC*), oFR6 (first codon of *gerAA*) – amplifying together the upstream region of gerAA; (2) oFR7 (containing the stop codon of *gerAC* and the end of *liaR* gene), oFR8 (*liaS*) – amplifying the downstream region of GerAA; and (3) oJM028, oJM029 – cm^R cassette, amplified from the pWX465 plasmid (*loxP-cat*, laboratory stock). The cm^R cassette was looped out leaving in-frame scar by using the temperature-sensitive plasmid constitutively expressing Cre recombinase[4].

bLA204 [Δ*gerBB::lox72,* Δ*gerKB::lox72,* Δ*yfkT::lox72,* Δ*yndE::lox72,* Δ*gerA::lox72, lacA::spec*] was generated by transforming bLA201 with gDNA from BDR3362 [*lacA::spec*]. All markless deletions were confirmed by PCR.

bLA207 [Δ*gerBB::lox72,* Δ*gerKB::lox72,* Δ*yfkT::lox72,* Δ*yndE::lox72,* Δ*gerA::lox72, lacA::spec, ycgO::kan*] was generated by transforming bLA204 with gDNA from BDR4077 [*ycgO::kan*]. All markless deletions were confirmed by PCR.

bLA210 [Δ*gerBB::lox72,* Δ*gerKB::lox72,* Δ*yfkT::lox72,* Δ*yndE::lox72,* Δ*gerA::lox72, lacA::spec, ycgO::kan, yhdG::cat*] was generated by transforming bLA207 with gDNA from BDR2815 [*yhdG::cat*]. All markless deletions were confirmed by PCR.

bLA212 [Δ*gerBB,* Δ*gerKB,* Δ*yfkT,* Δ*yndE,* Δ*gerA, lacA::gerAC(erm), ycgO::kan, yhdG::cat*] was generated by transforming bLA210 with pLA023 [*lacA::PgerA-RBSgerAC-gerAC-erm*].

bLA215 [Δ*gerBB,* Δ*gerKB,* Δ*yfkT,* Δ*yndE,* Δ*gerA, lacA::gerAC(erm), ycgO::gerAB(spec), yhdG::cat*] was generated by transforming bLA212 [Δ*gerBB,* Δ*gerKB,* Δ*yfkT,* Δ*yndE,* Δ*gerA, lacA::PgerA-gerAC(erm), ycgO::kan, yhdG::cat*] with pLA013 [*ycgO::PgerA-RBSgerAB-gerAB (spec)*].

bLA216 [Δ*gerBB,* Δ*gerKB,* Δ*yfkT,* Δ*yndE,* Δ*gerA, lacA::gerAC(erm), ycgO::kan, yhdG::gerAA(tet)*] was generated by transforming bLA212 with pLA039 [*yhdG::PgerA-RBSgerAB-gerAA-lox-tet-lox*].

bLA219 [Δ*gerBB,* Δ*gerKB,* Δ*yfkT,* Δ*yndE,* Δ*gerA, lacA::gerAC(erm), ycgO::gerAB(spec), yhdG::gerAA(tet)*] was generated by transforming bLA216 with pLA013 [*ycgO::PgerA-RBSgerAB-gerAB-lox-spec-lox*]

bLA286 [Δ*gerBB,* Δ*gerKB,* Δ*yfkT,* Δ*yndE,* Δ*gerA, lacA::gerAC(erm), ycgO::gerAB*(G25A)*(spec), yhdG::gerAA(tet)*] was generated by transforming bLA216 with pLA099 [*ycgO::PgerA-RBSgerAB-gerAB*(G25A)-*lox-spec-lox*].

bLA287 [Δ*gerBB,* Δ*gerKB,* Δ*yfkT,* Δ*yndE,* Δ*gerA, lacA::gerAC(erm), ycgO::gerAB*(V101A)*(spec), yhdG::gerAA(tet)]* was generated by transforming bLA216 with pLA100 [*ycgO::PgerA-RBSgerAB-gerAB*(V101A)-*lox-spec-lox*].

bLA289 [Δ*gerBB,* Δ*gerKB,* Δ*yfkT,* Δ*yndE,* Δ*gerA, lacA::gerAC(erm), ycgO::gerAB*(V101C)*(spec), yhdG::gerAA(tet)*] was generated by transforming bLA216 with pLA102 [*ycgO::PgerA-RBSgerAB-gerAB*(V101C)-*lox-spec-lox*]

bLA291 [Δ*gerBB,* Δ*gerKB,* Δ*yfkT,* Δ*yndE,* Δ*gerA, lacA::gerAC(erm), ycgO::gerAB*(G200A)*(spec), yhdG::gerAA(tet)*] was generated by transforming bLA216 with pLA104 [*ycgO::PgerA-RBSgerAB-gerAB*(G200A)-*lox-spec-lox*].

bLA292 [Δ*gerBB,* Δ*gerKB,* Δ*yfkT,* Δ*yndE,* Δ*gerA, lacA::gerAC(erm), ycgO::gerAB*(Y291S)*(spec), yhdG::gerAA(tet)*] was generated by transforming bLA216 with pLA105 [*ycgO::PgerA-RBSgerAB-gerAB*(Y291S)-*lox-spec-lox*]

bLA295 [Δ*gerBB,* Δ*gerKB,* Δ*yfkT,* Δ*yndE,* Δ*gerA, lacA::gerAC(erm), ycgO::gerAB*(L199I)*(spec), yhdG::gerAA(tet)*] was generated by transforming bLA216 with pLA120 [*ycgO::PgerA-RBSgerAB-gerAB*(L199I)-*lox-spec-lox*].

bLA296 [Δ*gerBB,* Δ*gerKB,* Δ*yfkT,* Δ*yndE,* Δ*gerA, lacA::gerAC(erm), ycgO::gerAB*(L199S)*(spec), yhdG::gerAA(tet)*] was generated by transforming bLA216 with pLA121 [*ycgO::PgerA-RBSgerAB-gerAB*(L199S)*-lox-spec-lox*].

bLA303 [Δ*gerBB,* Δ*gerKB,* Δ*yfkT,* Δ*yndE,* Δ*gerA, lacA::PgerA-gerAC(erm), ycgO::gerAB*(T287V)*(spec), yhdG::gerAA(tet)*] was generated by transforming bLA216 with pLA124 [*ycgO::PgerA-RBSgerAB-gerAB*(T287V)*-lox-spec-lox*].

bLA304 [Δ*gerBB,* Δ*gerKB,* Δ*yfkT,* Δ*yndE,* Δ*gerA, lacA::gerAC(erm), ycgO::gerAB*[T287L]*(spec), yhdG::gerAA(tet)*] was generated by transforming bLA216 with pLA125 [*ycgO::PgerA-RBSgerAB-gerAB*[T287L]*-lox-spec*].

bLA308 [Δ*gerBB,* Δ*gerKB,* Δ*yfkT,* Δ*yndE,* Δ*gerA, lacA::gerAC(erm), ycgO::gerAB*(V101F)*(spec), yhdG::gerAA(tet)*] was generated by transforming bLA216 with pLA129 [*ycgO::PgerA-RBSgerAB-gerAB*(V101F)*-lox-spec-lox*].

bLA315 [Δ*gerBB,* Δ*gerKB,* Δ*yfkT,* Δ*yndE,* Δ*gerA, lacA::gerAC(erm), ycgO::gerAB*(T287L)*(spec), yhdG::gerAA(tet), sleB::kan*] was generated by transforming *B. subtilis* bLA304 with an isothermal assembly product *sleB::kan* generated by three PCR products: The PCR products were amplified with: (1) oLA284 (*ypdA*), oLA283 (first codon of *sleB*) – amplifying together the upstream region of *sleB*; (2) oLA285 (containing the stop codon of *sleB* and the intergenic region between *sleB* and *ypeB*), oLA286 (*ypeB*) – amplifying the downstream region of *sleB*; and (3) oJM028, oJM029 – kan^R cassette, amplified from the pWX470 plasmid [*kan*] (laboratory stock).

bLA316 [Δ*gerBB,* Δ*gerKB,* Δ*yfkT,* Δ*yndE,* Δ*gerA, lacA::gerAC(erm), ycgO::gerAB*(V101F)(*spec*)*, yhdG:: gerAA(tet), sleB::kan*] was generated by transforming *B. subtilis* bLA308 with the isothermal assembly product *sleB::kan* as described for bLA315.

bLA321 [Δ*gerBB,* Δ*gerKB,* Δ*yfkT,* Δ*yndE,* Δ*gerA, lacA::gerAC-His (phleo), ycgO::gerAB(spec), yhdG::gerAA(tet)*] was generated by transforming bLA219 with pLA131 [*lacA::PgerA-RBSgerAC-gerAC-His-(phleo)*].

bLA322 [Δ*gerBB,* Δ*gerKB,* Δ*yfkT,* Δ*yndE,* Δ*gerA, lacA::gerAC-His (phleo), ycgO::kan, yhdG::gerAA(tet)*] was generated by transforming bLA216 with pLA131 [*lacA::PgerA-RBSgerAC-gerAC-His-(phleo)*].

bLA323 [Δ*gerBB,* Δ*gerKB,* Δ*yfkT,* Δ*yndE,* Δ*gerA, lacA::gerAC-His(phleo), ycgO::gerAB*(G25A)*(spec), yhdG::gerAA(tet)*] was generated by transforming bLA286 with pLA131 [*lacA::PgerA-RBSgerAC-gerAC-His(phleo)*].

bLA324 [Δ*gerBB,* Δ*gerKB,* Δ*yfkT,* Δ*yndE,* Δ*gerA, lacA::gerAC-His(phleo), ycgO::gerAB*(G200A)*(spec), yhdG::gerAA(tet)*] was generated by transforming bLA291 with pLA131 [*lacA::PgerA-RBSgerAC-gerAC-His(phleo)*].

bLA325 [Δ*gerBB,* Δ*gerKB,* Δ*yfkT,* Δ*yndE,* Δ*gerA, lacA::gerAC(erm), ycgO::gerAB*(G25V)*(spec), yhdG::gerAA(tet)*] was generated by transforming bLA216 with pLA132 [*ycgO::PgerA-RBSgerAB-gerAB*(G25V)*-lox-spec-lox*].

bLA326 [Δ*gerBB,* Δ*gerKB,* Δ*yfkT,* Δ*yndE,* Δ*gerA, lacA::gerAC-His(phleo), ycgO::gerAB*(G25V)*(spec), yhdG::gerAA(tet)*] was generated by transforming bLA325 [Δ*gerBB,* Δ*gerKB,* Δ*yfkT,* Δ*yndE* Δ*gerA, lacA::gerAC(erm), ycgO::gerAB*(G25V)*-lox-spec-lox, yhdG::gerAA-lox-tet*] with pLA131 [*lacA::PgerA-RBSgerAC-gerAC-His(phleo)*].

bLA328 [Δ*gerBB,* Δ*gerKB,* Δ*yfkT,* Δ*yndE, ΔgerA, lacA::PgerA-gerAC(erm), ycgO::PgerA-gerAB(spec), yhdG::PgerA-gerAA*(C100S*)(tet)*] was generated by transforming bLA215 [Δ*gerBB,* Δ*gerKB,* Δ*yfkT,* Δ*yndE, ΔgerA, lacA::gerAC(erm), ycgO::gerAB(spec), yhdG::cat*] with pLA137 [*yhdG::PgerA-RBSgerAB-gerAA*(C100S)(*tet)*].

bLA335 [Δ*gerBB,* Δ*gerKB,* Δ*yfkT,* Δ*yndE, ΔgerA, lacA::PgerA-gerAC(erm), ycgO::gerAB(spec), yhdG:: gerAA*(S55C, C100S)*(tet)*] was generated by transforming bLA215 [Δ*gerBB,* Δ*gerKB,* Δ*yfkT,* Δ*yndE, ΔgerA, lacA::gerAC(erm), ycgO::gerAB(spec), yhdG::cat*] with pLA138 [*yhdG::PgerA-RBSgerAB-gerAA*(S55C, C100S)*(tet)*].

bLA336 [Δ*gerBB,* Δ*gerKB,* Δ*yfkT,* Δ*yndE, ΔgerA, lacA::gerAC(erm), ycgO::PgerA-gerAB(spec), yhdG::PgerA-gerAA*(S94C, C100S)*(tet)*] was generated by transforming bLA215 [Δ*gerBB,* Δ*gerKB,* Δ*yfkT,* Δ*yndE, ΔgerA, lacA::gerAC(erm), ycgO::gerAB(spec), yhdG::cat*] with pLA139 [*yhdG::PgerA-RBSgerAB-gerAA*(S94C, C100S)].

bLA338 [Δ*gerBB,* Δ*gerKB,* Δ*yfkT,* Δ*yndE, ΔgerA, lacA::gerAC(erm), ycgO::gerAB(spec), yhdG::gerAA*(C100S, S317C)*(tet)*] was generated by transforming bLA215 [Δ*gerBB,* Δ*gerKB,* Δ*yfkT,* Δ*yndE, ΔgerA, lacA::gerAC(erm), ycgO::gerAB(spec), yhdG::cat*] with pLA141 [*yhdG::PgerA-RBSgerAB-gerAA*(C100S, S317C)(*tet)*].

bLA343 [Δ*gerBB,* Δ*gerKB,* Δ*yfkT,* Δ*yndE, ΔgerA, lacA::spec, ycgO::kan, yhdG::Pveg-gerAA*(C100S)*(tet)*] was generated by transforming bLA210 [Δ*gerBB,* Δ*gerKB,* Δ*yfkT,* Δ*yndE,* Δ*gerA, lacA::spec, ycgO::kan, yhdG::cat*] with pLA150 [*yhdG::Pveg-gerAA*(C100S)*(tet)*].

bLA344 [Δ*gerBB,* Δ*gerKB,* Δ*yfkT,* Δ*yndE, ΔgerA, lacA::spec, ycgO::kan, yhdG::Pveg-gerAA*(S55C, C100S)*(tet)*]was generated by transforming bLA210 [Δ*gerBB,* Δ*gerKB,* Δ*yfkT,* Δ*yndE,* Δ*gerA, lacA::spec, ycgO::kan, yhdG::cat*] with pLA151 [*yhdG::Pveg-gerAA*(S55C, C100S)*(tet)*].

bLA345 [Δ*gerBB,* Δ*gerKB,* Δ*yfkT,* Δ*yndE, ΔgerA, lacA::spec, ycgO::kan, yhdG::Pveg-gerAA*(S94C, C100S)(*tet)*] was generated by transforming bLA210 [Δ*gerBB,* Δ*gerKB,* Δ*yfkT,* Δ*yndE,* Δ*gerA, lacA::spec, ycgO::kan, yhdG::cat*] with pLA152 [*yhdG::Pveg-gerAA*(S94C, C100S)*(tet)*].

bLA347 [Δ*gerBB,* Δ*gerKB,* Δ*yfkT,* Δ*yndE, ΔgerA, lacA::spec, ycgO::kan, yhdG::Pveg-gerAA*(C100S, S317)*(tet)*] was generated by transforming bLA210 [Δ*gerBB,* Δ*gerKB,* Δ*yfkT,* Δ*yndE,* Δ*gerA, lacA::spec, ycgO::kan, yhdG::cat*] with pLA154 [*yhdG::Pveg-gerAA*(C100S, S317C)(*tet)*].

bLA353 [Δ*gerBB,* Δ*gerKB,* Δ*yfkT,* Δ*yndE, ΔgerA, lacA::Pveg-gerAC-His(phleo), ycgO::kan, yhdG::Pveg-gerAA*(C100S)*(tet)*] was generated by transforming bLA343 [Δ*gerBB,* Δ*gerKB,* Δ*yfkT,* Δ*yndE, ΔgerA, lacA::spec, ycgO::kan, yhdG::Pveg-gerAA*(C100S)(*tet)*] with pLA156 [*lacA::Pveg -gerAC-His(phleo)*].

bLA354 [Δ*gerBB,* Δ*gerKB,* Δ*yfkT,* Δ*yndE, ΔgerA, lacA::Pveg-gerAC-His(phleo), ycgO::kan, yhdG::Pveg-gerAA*(S55C, C100S)*(tet)*] was generated by transforming bLA344 [Δ*gerBB,* Δ*gerKB,* Δ*yfkT,* Δ*yndE, ΔgerA, lacA::spec, ycgO::kan, yhdG::Pveg-gerAA*(S55C, C100S)*(tet)*] with pLA156 [*lacA::Pveg -gerAC-His(phleo)*].

bLA355 [Δ*gerBB,* Δ*gerKB,* Δ*yfkT,* Δ*yndE, ΔgerA, lacA::Pveg-gerAC-His(phleo), ycgO::kan, yhdG::Pveg-gerAA*(S94C, C100S)*(tet)*] was generated by transforming bLA345 [Δ*gerBB,* Δ*gerKB,* Δ*yfkT,* Δ*yndE, ΔgerA, lacA::spec, ycgO::kan, yhdG::Pveg-gerAA*(S94C, C100S)(*tet)*] with pLA156 [*lacA::Pveg -gerAC-His(phleo)*].

bLA357 [Δ*gerBB,* Δ*gerKB,* Δ*yfkT,* Δ*yndE, ΔgerA, lacA::Pveg-gerAC-His(phleo), ycgO::kan, yhdG::Pveg-gerAA*(C100S, S317C)*(tet)*] was generated by transforming bLA347 [Δ*gerBB,* Δ*gerKB,* Δ*yfkT,* Δ*yndE, ΔgerA, lacA::spec, ycgO::kan, yhdG::Pveg-gerAA*(C100S, S317)*(tet)*] with pLA156 [*lacA::Pveg -gerAC-His(phleo)*].

bLA358 [Δ*gerBB,* Δ*gerKB,* Δ*yfkT*, Δ*yndE*, Δ*gerA*, *lacA::Pveg- gerAC-His(phleo), ycgO::Pveg-gerAB(spec), yhdG::Pveg-gerAA*(C100S)*(tet)]* was generated by transforming bLA353 [Δ*gerBB,* Δ*gerKB,* Δ*yfkT,* Δ*yndE, ΔgerA, lacA::Pveg- gerAC-His(phleo), ycgO::kan, yhdG::Pveg-gerAA*(C100S) *(tet)*] with pLA155 [*ycgO::Pveg-gerAB(spec)*].

bLA359 [Δ*gerBB,* Δ*gerKB,* Δ*yfkT,* Δ*yndE, ΔgerA, lacA::Pveg-gerAC-His(phleo), ycgO::Pveg-gerAB(spec), yhdG::Pveg-gerAA(S55C, C100S)(tet)*] was generated by transforming bLA354 [Δ*gerBB,* Δ*gerKB,* Δ*yfkT,* Δ*yndE, ΔgerA, lacA::Pveg-gerAC-His(phleo), ycgO::kan, yhdG::Pveg-gerAA*(S55C, C100S)*(tet)*] with pLA155 [*ycgO::Pveg-gerAB(spec)*].

bLA360 [Δ*gerBB,* Δ*gerKB,* Δ*yfkT,* Δ*yndE, ΔgerA, lacA::Pveg-gerAC-His(phleo), ycgO::Pveg-gerAB(spec), yhdG::Pveg-gerAA*(S94C, C100S)*(tet)*] was generated by transforming bLA355 [Δ*gerBB,* Δ*gerKB,* Δ*yfkT,* Δ*yndE, ΔgerA, lacA::Pveg-gerAC-His(phleo), ycgO::kan, yhdG::Pveg-gerAA*(S94C, C100S)*(tet)*] with pLA155 [*ycgO::Pveg-gerAB(spec)*].

bLA362 [Δ*gerBB,* Δ*gerKB,* Δ*yfkT,* Δ*yndE, ΔgerA, lacA::Pveg-gerAC-His(phleo), ycgO::Pveg-gerAB(spec), yhdG::Pveg-gerAA(C100S, S317C)(tet)*] was generated by transforming bLA357 [Δ*gerBB,* Δ*gerKB,* Δ*yfkT,* Δ*yndE, ΔgerA, lacA::Pveg-gerAC-His(phleo), ycgO::kan, yhdG::Pveg-gerAA*(C100S, S317C)*(tet)*] with pLA155 [*ycgO::Pveg-gerAB(spec)*].

bLA363 [Δ*gerBB,* Δ*gerKB,* Δ*yfkT,* Δ*yndE, ΔgerA, lacA::Pveg-gerAC-His(phleo), ycgO::Pveg-gerAB(spec), yhdG::Pveg-gerAA*(C100S)(*tet), amyE::PxylA-gfp-spoIVFA(cat)*] was generated by transforming bLA358 [Δ*gerBB,* Δ*gerKB,* Δ*yfkT*, Δ*yndE*, Δ*gerA*, *lacA::Pveg-gerAC-His(phleo), ycgO::Pveg-gerAB(spec), yhdG::Pveg-gerAA*(C100S)*(tet)*] with pDR124 [*amyE::PxylA-gfp-spoIVFA(cat)*].

bLA368 [Δ*gerBB,* Δ*gerKB,* Δ*yfkT,* Δ*yndE,* Δ*gerA, lacA::PgerA-gerAC-His(phleo), ycgO::PgerA-gerAB*(V101A)*(spec), yhdG::PgerA-gerAA(tet)*] was generated by transforming bLA287 [Δ*gerBB,* Δ*gerKB,* Δ*yfkT,* Δ*yndE,* Δ*gerA, lacA::PgerA-gerAC(erm), ycgO::PgerA-gerAB*(V101A)*(spec), yhdG::PgerA-gerAA(tet)*] with pLA131 [*lacA::PgerA-RBSgerAC-gerAC-His(phleo)*].

bLA369 [Δ*gerBB,* Δ*gerKB,* Δ*yfkT,* Δ*yndE,* Δ*gerA, lacA::PgerA-gerAC-His(phleo), ycgO::PgerA-gerAB*(V101C)*(spec), yhdG::PgerA-gerAA(tet)*] was generated by transforming bLA289 [Δ*gerBB,* Δ*gerKB,* Δ*yfkT,* Δ*yndE,* Δ*gerA, lacA::PgerA-gerAC(erm), ycgO::PgerA-gerAB*(V101C)*(spec), yhdG::PgerA-gerAA(tet)*] with pLA131 [*lacA::PgerA-RBSgerAC-gerAC-His(phleo)*].

bLA370 [Δ*gerBB,* Δ*gerKB,* Δ*yfkT,* Δ*yndE,* Δ*gerA, lacA::PgerA-gerAC-His(phleo), ycgO::PgerA-gerAB*(Y291S)*(spec), yhdG::PgerA-gerAA(tet)*] was generated by transforming bLA292 [Δ*gerBB,* Δ*gerKB,* Δ*yfkT,* Δ*yndE,* Δ*gerA, lacA::PgerA-gerAC(erm), ycgO::PgerA-gerAB*(Y291S)*(spec), yhdG::PgerA-gerAA(tet)*] with pLA131 [*lacA::PgerA-RBSgerAC-gerAC-His(phleo)*].

bLA371 [Δ*gerBB,* Δ*gerKB,* Δ*yfkT,* Δ*yndE,* Δ*gerA, lacA::PgerA-gerAC-His(phleo), ycgO::PgerA-gerAB*(L199I)*(spec), yhdG::PgerA-gerAA(tet)*] was generated by transforming bLA295 [Δ*gerBB,* Δ*gerKB,* Δ*yfkT,* Δ*yndE,* Δ*gerA, lacA::PgerA-gerAC(erm), ycgO::PgerA-gerAB*(L199I)*(spec), yhdG::PgerA-gerAA(tet)*] with pLA131 [*lacA::PgerA-RBSgerAC-gerAC-His(phleo)*].

bLA372 [Δ*gerBB,* Δ*gerKB,* Δ*yfkT,* Δ*yndE,* Δ*gerA, lacA::PgerA-gerAC-His(phleo), ycgO::PgerA-gerAB*(L199S)*(spec), yhdG::PgerA-gerAA(tet)*] was generated by transforming bLA296 [Δ*gerBB,* Δ*gerKB,* Δ*yfkT,* Δ*yndE,* Δ*gerA, lacA::PgerA-gerAC(erm), ycgO::PgerA-gerAB*(L199S)*(spec), yhdG::PgerA-gerAA(tet)*] with pLA131 [*lacA::PgerA-RBSgerAC-gerAC-His(phleo)*].

bLA373 [Δ*gerBB,* Δ*gerKB,* Δ*yfkT,* Δ*yndE,* Δ*gerA, lacA::PgerA-gerAC-His(phleo), ycgO::PgerA-gerAB*(T287V)*(spec), yhdG::PgerA-gerAA(tet)*] was generated by transforming bLA303 [Δ*gerBB,* Δ*gerKB,* Δ*yfkT,* Δ*yndE,* Δ*gerA, lacA::PgerA-gerAC(erm), ycgO::PgerA-gerAB*(T287V)*(spec), yhdG::PgerA-gerAA(tet)*] with pLA131 [*lacA::PgerA-RBSgerAC-gerAC-His(phleo)*].

bLA374 [Δ*gerBB,* Δ*gerKB,* Δ*yfkT,* Δ*yndE,* Δ*gerA, lacA::PgerA-gerAC-His(phleo), ycgO::PgerA-gerAB*(**T287L**)(*spec), yhdG::PgerA-gerAA(tet), sleB::kan*] was generated by transforming bLA315 [Δ*gerBB,* Δ*gerKB,* Δ*yfkT,* Δ*yndE,* Δ*gerA, lacA::PgerA-gerAC(erm), ycgO::PgerA-gerAB*(T287L)(*spec), yhdG::PgerA-gerAA(tet), sleB::kan*] with pLA131 [*lacA::PgerA-RBSgerAC-gerAC-His(phleo)*].

bLA375 [Δ*gerBB,* Δ*gerKB,* Δ*yfkT,* Δ*yndE,* Δ*gerA, lacA::PgerA-gerAC-His(phleo), ycgO::PgerA-gerAB*(V101F)(*spec), yhdG::PgerA-gerAA(tet), sleB::kan*] was generated by transforming bLA316 [Δ*gerBB,* Δ*gerKB,* Δ*yfkT,* Δ*yndE,* Δ*gerA, lacA::PgerA-gerAC(erm), ycgO::PgerA-gerAB*(V101F)*(spec), yhdG::PgerA-gerAA(tet), sleB::kan*] with pLA131 [*lacA::PgerA-RBSgerAC-gerAC-His(phleo)*].

bLA376 [Δ*gerBB,* Δ*gerKB,* Δ*yfkT,* Δ*yndE,* Δ*gerA, lacA::PgerA-gerAC-His(phleo), ycgO::PgerA-gerAB(spec), yhdG::PgerA-gerAA(tet), sleB::kan*] was generated by transforming *B. subtilis* bLA321 with the isothermal assembly product *sleB::kan* as described for bLA315.

Plasmid constructions

pLA010 [*ycgO::PgerA (spec)*] was constructed in a two-way ligation with an EcoRI-SpeI *PgerA* PCR product, amplified with oLA041and oLA042 from *B. subtilis* 168 genomic DNA, and pCB014 [*ycgO::spec*] cut with EcoRI and SpeI. pCB014 is a double-crossover integration vector at the *ycgO* locus with a $spec^R$ cassette (laboratory stock).

pLA013 [*ycgO::PgerA-RBSgerAB-gerAB (spec)*] was constructed in a two-way ligation with a SpeI-BamHI *gerAB* PCR product, amplified with oLA046 and oLA047 from *B. subtilis* 168 genomic DNA, and pLA010 [*ycgO::PgerA (spec)*] cut with SpeI and BamHI.

pLA023 [*lacA::PgerA-RBSgerAC-gerAC (erm)*] was constructed in a two-way ligation with an XhoI-BamHI *PgerA-RBSgerAC-gerAC* PCR product, amplified with oLA049 and oLA089 from pLA014 [*ycgO::PgerA-RBSgerAC-gerAC-(spec)*], and pDR183 [*lacA::erm*], cut with XhoI and BamHI. pLA014 was constructed in a two-way ligation with SpeI-BamHI *RBSgerAC-gerAC* PCR product, amplified with oLA048, oLA049 from *B. subtilis* 168 genomic DNA, and pLA010 [*ycgO::PgerA (spec)*], cut with SpeI and BamHI. pDR183 is a double-crossover integration vector at the *lacA* locus with an erm^R cassette (laboratory stock).

pLA039 [*yhdG::PgerA-RBSgerAB-gerAA (tet)*] was constructed in a two-way ligation with an EcoRI-BamHI *PgerA-RBSgerAB-gerAA* PCR product amplified with oLA041 and oLA112, amplified from pLA029 [*ycgO::PgerA-RBSgerAB-gerAA (spec)*], and pCB012 [*yhdG::tet*], cut with EcoRI and BamHI. pLA029 was constructed in a two-way ligation with a SpeI-BamHI *RBS-gerAB-gerAA* PCR product amplified with oLA103 and oLA112, amplified from *B. subtilis* 168 genomic DNA, and pLA010 [*ycgO::PgerA(spec)*], restricted with SpeI and BamHI. pCB012 is a double-crossover integration vector at the *yhdG* locus with a tet^R cassette (laboratory stock).

pLA099 [*ycgO::PgerA-RBSgerAB-gerAB*(G25A) *(spec)*] was constructed by site-directed mutagenesis using oligonucleotide oLA197 and plasmid pLA013.

pLA100 [*ycgO::PgerA-RBSgerAB-gerAB*(V101A) *(spec)*] was constructed by site-directed mutagenesis using oligonucleotide oLA198 and plasmid pLA013.

pLA102 [*ycgO::PgerA-RBSgerAB-gerAB*(V101C) *(spec)*] was constructed by site-directed mutagenesis using oligonucleotide oLA200 and plasmid pLA013.

pLA104 [*ycgO::PgerA-RBSgerAB-gerAB*(G200A*) (spec)*] was constructed by site-directed mutagenesis using oligonucleotide oLA202 and plasmid pLA013.

pLA105 [*ycgO::PgerA-RBSgerAB-gerAB*(Y291S) *(spec)*] was constructed by site-directed mutagenesis using oligonucleotide oLA203 and plasmid pLA013.

pLA120 [*ycgO::PgerA-RBSgerAB-gerAB*(L199I) *(spec)*] was constructed by site-directed mutagenesis using oligonucleotide oLA293 and plasmid pLA013.

pLA121 [*ycgO::PgerA-RBSgerAB-gerAB*(L199S) *(spec)*] was constructed by site-directed mutagenesis using oligonucleotide oLA294 and plasmid pLA013

pLA124 [*ycgO::PgerA-RBSgerAB-gerAB*(T287V) *(spec)*] was constructed by site-directed mutagenesis using oligonucleotide oLA297 and plasmid pLA013.

pLA125 [*ycgO::PgerA-RBSgerAB-gerAB*(T287L) *(spec)*] was constructed by site-directed mutagenesis using oligonucleotide oLA298 and plasmid pLA013.

pLA129 [*ycgO::PgerA-RBSgerAB-gerAB*(V101F) *(spec)*] was constructed by site-directed mutagenesis using oligonucleotide oLA302 and plasmid pLA013.

pLA131 [*lacA::PgerA-RBSgerAC-gerAC-His6 (phleo)*] was constructed in a two-way ligation with an XhoI-BamHI PCR product containing *PgerA-RBSgerAC-gerAC-His6* amplified with oLA89 and oLA308 and pNC014 [*lacA::phleo*]. pNC014 is a double-crossover integration vector that contains a $phleo^R$ cassette (laboratory stock).

pLA137 [*yhdG::PgerA-RBSgerAB-gerAA*(C100S) *(tet)*] was constructed by site-directed mutagenesis using oligonucleotide oLA323 and plasmid pLA039

pLA138 [*yhdG::PgerA-RBSgerAB-gerAA*(S55C, C100S) *(tet)*] was constructed by site-directed mutagenesis using oligonucleotide oLA324 and plasmid pLA137

pLA139 [*yhdG::PgerA-RBSgerAB-gerAA*(S94C, C100S) *(tet)*] was constructed by site-directed mutagenesis using oligonucleotide oLA340 and plasmid pLA137

pLA141 [*yhdG::PgerA-RBSgerAB-gerAA*(C100S, S317C)*(tet)*] was constructed by site-directed mutagenesis using oligonucleotide oLA327 and plasmid pLA137

pLA150 [*yhdG::Pveg-RBSgerAB-gerAA*(C100S)*(tet)*] was constructed by two-way ligation with a EcoRI-SpeI *Pveg* PCR product amplified with oLA359 and oLA360, amplified from pER075 [*sacA::Pveg-mCherry(kan)*] (laboratory stock), and pLA137 [*yhdG::PgerA-RBSgerAB-gerAA*(C100S)*(tet)*], cut with EcoRI and SpeI.

pLA151 [*yhdG::Pveg-RBSgerAB-gerAA*(S55C, C100S)*(tet)*] was constructed by two-way ligation with a EcoRI-SpeI *Pveg* PCR product amplified with oLA359 and oLA360, amplified from pER075 [*sacA::Pveg-mCherry(kan)*] (laboratory stock), and pLA138 [*yhdG::PgerA-RBSgerAB-gerAA*(S55C, C100S)*(tet)*], cut with EcoRI and SpeI.

pLA152 [*yhdG::Pveg-RBSgerAB-gerAA*(S94C, C100S)*(tet)*] was constructed by two-way ligation with a EcoRI-SpeI *Pveg* PCR product amplified with oLA359 and oLA360, amplified from pER075 [*sacA::Pveg-mCherry(kan)*] (laboratory stock) and pLA139 [*yhdG::PgerA-RBSgerAB-gerAA*(S94C, C100S)*(tet)*], cut with EcoRI and SpeI.

pLA154 [*yhdG::Pveg-RBSgerAB-gerAA*(C100S, S317C)*(tet)*] was constructed by two-way ligation with a EcoRI-SpeI *Pveg* PCR product amplified with oLA359 and oLA360, amplified from pER075 [*sacA::Pveg-mCherry(kan)*] (laboratory stock), and pLA141 [*yhdG::PgerA-RBSgerAB-gerAA*(C100S, S317C)*(tet)*], cut with EcoRI and SpeI.

pLA155 [*ycgO::Pveg-RBSgerAB-gerAB(spec)*] was constructed by two-way ligation with a EcoRI-SpeI *Pveg* PCR product amplified with oLA359 and oLA360, amplified from pER075 [*sacA::Pveg-mCherry(kan)*](laboratory stock), and pLA013 [*ycgO::PgerA-RBSgerAB-gerAB(spec)*], cut with EcoRI and SpeI.

pLA156 [*lacA::Pveg-RBSgerAC-gerAC-His(phleo)*] was constructed by two-way ligation with a XhoI-SpeI *Pveg* PCR product amplified with oLA365 and oLA360, amplified from pLA150 [*yhdG::Pveg-RBSgerAB-gerAA*[C100S]*(tet)*], and pLA131 [*lacA::PgerA-RBSgerAC-gerAC-His(phleo)*], cut with XhoI and SpeI.

Substituted-Cysteine Accessibility Method (SCAM)

SCAM assays were performed with protoplasts of vegetatively growing *B. subtilis* cells expressing functional GerAA variants (**Supplementary Table 1**) with GerAB and GerAC under the control of the P*veg* promoter. Protoplasts were required due to sulfhydryl reactive groups in the cell envelope that prevented efficient crosslinking of cysteines in the extracytoplasmic domains of membrane proteins. All four strains contained a functional GerAA variant (C100S) that lacked endogenous cysteine residues. Pre-cultures of strains expressing GerAA(C100S) (bLA358), GerAA(S55C, C100S) (bLA359), GerAA(S94C, C100S) (bLA360), and GerAA(C100S, S317C) (bLA362) were grown in 3 ml LB at 37˚C to an OD_{600} of ~0.5 and then used to inoculate 20 mL of LB to OD_{600}=0.025. Cultures were grown at 37˚C to OD_{600} of 0.6 and then 5 mL from each were centrifuged (10000 x g, 2 min). Cell pellets were resuspended in 1 mL of 1XSMM (0.5 M sucrose, 20 mM $MgCl_2$, 20 mM maleic acid pH 6.5)[5] supplemented with lysozyme at a final concentration of 2 mg/mL. The suspensions were incubated at room temperature for 25 min with rotation until >95% of the cells had converted to protoplasts as assessed by phase-contrast microscopy. The protoplasts were collected by centrifugation (2300 x g, 10 min), washed once with 1XSMM lacking lysozyme, and resuspended in 500 µL 1XSMM. 100 µL aliquots of protoplasts were used in five reactions. To reaction 1, 100 µL of 20 mM MTSES (2-Sulfonatoethyl methanethiosulfonate sodium salt, Biotium, dissolved in 1XSMM) was added to block extracytoplasmic cysteines (final concentration of 10 mM). To reactions 2, 3 and 4, 100 µL of 1XSMM was added. To reaction 5, 100 µl of 4 mM N-ethylmaleimide (NEM) was added to block both cytoplasmic and extracytoplasmic cysteines (final concentration of 2 mM). Reactions 1, 2, and 3 were incubated at room temperature for 10 min with rotation followed by the addition of 22 µL 0.3 M L-cysteine (dissolved in 1XSMM) to quench the MTSES. Protoplasts were incubated at RT for an additional 10 min with rotation. Reactions 4 and 5 were incubated for 100 min with rotation followed by quenching with L-cysteine as mentioned for reactions 1, 2, and 3. The protoplasts were then washed three times with 1XSMM. Washed protoplasts were resuspended in 100 µL PEGylation buffer (100 mM Tris-HCl, pH 7.5, 1 mM EDTA, 1% SDS, 10 M Urea). To reactions 1, 2, 4 and 5, 20 µL 1.2 mM mPEG-Mal (monofunctional maleimide polyethylene glycol, molecular weight 5 kDa, Creative PEGWorks) was added (final concentration 200 µM). To reaction 3, 20 µL of ddH_2O was added. Samples were incubated at 30˚C for 30 min in the dark with rotation. Samples were precipitated with 10% TCA (Trichloroacetic acid) to remove excess mPEG-MAL. Protein pellets were collected by centrifugation (20000 x g, 30 min at 4˚C), washed with 1 ml of ice-cold acetone, air-dried and resuspended in 100 µL 2X sample buffer (0.25 M Tris pH 6.8, 4% SDS, 20% glycerol, 10 mM EDTA. 10% β-Mercaptoethanol). Proteins were resolved by SDS-PAGE followed by immunoblot with anti-GerAA[6] (1:5,000) and anti-σ^{A}[7] (1:10,000) antibodies.

Protease accessibility assay

Protease accessibility assays were performed on protoplasts of vegetatively growing *B. subtilis* cells expressing GerAA, GerAB, and GerAC under the control of the P*veg* promoter and the sporulation membrane protein GFP-SpoIVFA under control of the xylose-regulated P*xylA* promoter. A pre-culture of strain bLA363 was grown in 3 ml LB at 37˚C to an OD_{600} of ~0.5 and then used to inoculate 25 mL of LB supplemented with 1 mM xylose to OD_{600}=0.025. Cultures were grown at 37˚C to OD_{600} of 0.5. Cells were collected by centrifugation (8K rpm, 5 min), and washed once with 1XSMM. Cells were resuspended in 5 mL 1XSMM supplemented with lysozyme (2 mg/mL final) and incubated at room temperature for 25 min with rotation until >95% of the cells had converted to protoplasts as assessed by phase-contrast microscopy. The protoplasts were collected by centrifugation (2300 x g, 10 min), washed once with 1XSMM and resuspended in 1 mL 1XSMM. Protoplasts were distributed into five aliquots (200 µL each) and treated with proteinase K (NEB, final concentration 50 µg/mL) for 0, 5, 10, and 20 minutes, or 20 minutes incubation with no proteinase K, followed by the addition of phenylmethylsulfonyl fluoride (PMSF) (5 mM final) to inactivate the protease. 2X sample buffer (0.25 M Tris pH 6.8, 4% SDS, 20% glycerol, 10 mM EDTA 10% β-Mercaptoethanol) was added to each reaction to a final volume of 500 µL. Proteins were resolved by SDS-PAGE followed by immunoblot analysis using anti-GerAA[6] (1:5000), anti-EzrA[8] (1:10,000), anti-ScpB[9] (1:10,000), anti-SpoIVFA[10] (1:10,000), and anti-GFP[11] (1:10,000) antibodies.

Evolutionary co-variation analysis supplement

For the GerAB monomer, we used a 2019 Uniref100 (https://www.uniprot.org/uniref/) database to build the alignment using a 5-iteration jackhmmer protocol with domain and sequence bitscore thresholds both set to 0.3*(the length of the protein). The resulting alignment primarily contains protein sequences with 'spore germination' in the description, but there are some proteins of unknown function and other annotations. Monomer evcouplings analysis was performed directly on the resulting alignment after removing sequences with more than 50% gaps compared to the *B. subtilis* GerAB sequence. Additionally, for the GerAB-GerAA complex interaction predictions only, we filtered the monomer alignments for these two proteins so that we were only considering sequences from species with GerAA and GerAB hits within 10,000 nucleotides of each other on the genome.

For species included in the GerAB monomer alignment, the overwhelming majority (19,986 out 20,525 sequences) are from Firmicutes. A small fraction from eukaryotes and other non-Firmicutes bacteria were recovered. The alignment did not have significant overlap with the GkApcT family based on domain analysis. The sequence for PDB 5OQT, a GkApcT transporter with Uniprot ID Q5L1G5 that was used to build the homology model for GerAB, was not present in the alignment used for the evcouplings analysis. Furthermore, the alignment sequences were mapped to 12,016 SwissProt + TREMBL IDs, which was then cross-referenced against the PFAM domain database to assess overlap between amino acid transporter domains and the alignment. All of the alignment sequences either have no domain annotated or have PFAM ID PF03845 (spore germination protein). No proteins annotated with PFAM ID PF13520 (amino acid permease), the domain associated with the GkApcT structure were detected.

References

1. Zeigler, D. R. *et al.* The origins of 168, W23, and other *Bacillus subtilis* legacy strains. *J. Bacteriol.* **190**, 6983–6995 (2008).
2. Ramírez-Guadiana, F. H., Meeske, A. J., Wang, X., Rodrigues, C. D. A. & Rudner, D. Z. The *Bacillus subtilis* germinant receptor GerA triggers premature germination in response to morphological defects during sporulation. *Mol. Microbiol.* **105**, 689–704 (2017).
3. Doan, T., Marquis, K. A. & Rudner, D. Z. Subcellular localization of a sporulation membrane protein is achieved through a network of interactions along and across the septum. *Mol. Microbiol.* **55**, 1767–81 (2005).
4. Meeske, A. J. *et al.* MurJ and a novel lipid II flippase are required for cell wall biogenesis in *Bacillus subtilis*. *Proc. Natl. Acad. Sci. U. S. A.* **112**, 6437–42 (2015).
5. Hardwood, C.R., Cutting, S. M. *Molecular biological methods for bacillus*. (Chichester; New York: Wiley, 1990).
6. Ramirez-Peralta, A., Zhang, P., Li, Y. qing & Setlow, P. Effects of sporulation conditions on the germination and germination protein levels of *Bacillus subtilis* spores. *Appl. Environ. Microbiol.* **78**, 2689–2697 (2012).
7. Fujita, M. & Sadaie, Y. Rapid isolation of RNA polymerase from sporulating cells of *Bacillus subtilis*. *Gene* **221**, 185–190 (1998).
8. Haeusser, D. P., Schwartz, R. L., Smith, A. M., Oates, M. E. & Levin, P. A. EzrA prevents aberrant cell division by modulating assembly of the cytoskeletal protein FtsZ. *Mol. Microbiol.* **52**, 801–814 (2004).
9. Wang, X., Tang, O. W., Riley, E. P. & Rudner, D. Z. The SMC condensin complex is required for origin segregation in *Bacillus subtilis*. *Curr. Biol.* **24**, 287–292 (2014).
10. Resnekov, O., Alper, S. & Losick, R. Subcellular localization of proteins governing the proteolytic activation of a developmental transcription factor in *Bacillus subtilis*. *Genes to Cells* **1**, 529–542 (1996).
11. Rudner, D. Z. & Losick, R. A sporulation membrane protein tethers the pro-σK processing enzyme to its inhibitor and dictates its subcellular localization. *Genes Dev.* **16**, 1007–1018 (2002).
