# Supplementary material for: Dormant spores sense amino acids through the B subunits of their germination receptors
Source: Nat Commun. 2021 Nov 25;12:6842. doi: 10.1038/s41467-021-27235-2 (PMC8617281; doi:10.1038/s41467-021-27235-2)
Supplement: Supplementary file 3 — Reporting Summary [file 41467_2021_27235_MOESM3_ESM.pdf]

nature research

Corresponding author(s): David Rudner

Last updated by author(s): Oct 7, 2021

Reporting Summary

Nature Research wishes to improve the reproducibility of the work that we publish. This form provides structure for consistency and transparency in reporting. For further information on Nature Research policies, see our Editorial Policies and the Editorial Policy Checklist.

Statistics

For all statistical analyses, confirm that the following items are present in the figure legend, table legend, main text, or Methods section.

n/a	Confirmed	
☒	☐	The exact sample size (n) for each experimental group/condition, given as a discrete number and unit of measurement
☐	☒	A statement on whether measurements were taken from distinct samples or whether the same sample was measured repeatedly
☒	☐	The statistical test(s) used AND whether they are one- or two-sided<br>*Only common tests should be described solely by name; describe more complex techniques in the Methods section.*
☒	☐	A description of all covariates tested
☒	☐	A description of any assumptions or corrections, such as tests of normality and adjustment for multiple comparisons
☐	☒	A full description of the statistical parameters including central tendency (e.g. means) or other basic estimates (e.g. regression coefficient) AND variation (e.g. standard deviation) or associated estimates of uncertainty (e.g. confidence intervals)
☒	☐	For null hypothesis testing, the test statistic (e.g. F, t, r) with confidence intervals, effect sizes, degrees of freedom and P value noted<br>*Give P values as exact values whenever suitable.*
☒	☐	For Bayesian analysis, information on the choice of priors and Markov chain Monte Carlo settings
☒	☐	For hierarchical and complex designs, identification of the appropriate level for tests and full reporting of outcomes
☒	☐	Estimates of effect sizes (e.g. Cohen's d, Pearson's r), indicating how they were calculated

Our web collection on statistics for biologists contains articles on many of the points above.

Software and code

Policy information about availability of computer code

Data collection	EVcouplings software version 0.0.5 (GerAA and GerAB) and 0.1.1 (GerVB) used was developed by the Marks lab and is publicly available at <https://github.com/debbiemarkslab/EVcouplings>. Alignments were built using Jackhmmer software (http://hmmer.org/download.html) against the Uniref100 sequence database downloaded January 2020 (GerAA and GerAB) or July 2020 (GerVB) (https://www.uniprot.org/help/uniref).
Data analysis	All EVcouplings software is available at <https://github.com/debbiemarkslab/EVcouplings>, as described in methods. As part of this pipeline, we used an optimized pseudolikelihood maximization package <https://github.com/debbiemarkslab/plmc> to identify strongly coupled residues based on the described alignments.<br>Microscopy images were analyzed using MetaMorph software (Molecular Devices; version 7.7)

For manuscripts utilizing custom algorithms or software that are central to the research but not yet described in published literature, software must be made available to editors and reviewers. We strongly encourage code deposition in a community repository (e.g. GitHub). See the Nature Research guidelines for submitting code & software for further information.

Data

Policy information about availability of data

All manuscripts must include a data availability statement. This statement should provide the following information, where applicable:

- Accession codes, unique identifiers, or web links for publicly available datasets
- A list of figures that have associated raw data
- A description of any restrictions on data availability

All datasets generated in this study are available at <https://github.com/debbiemarkslab/GerA-suppData-2021>. For EVcouplings analysis, all data for alignments were collected from publicly available datasets (Uniref100) (https://www.uniprot.org/uniref/) and ENA genome location tables shared on the Mark’s lab website:

<https://marks.hms.harvard.edu/evcomplex_databases/cds_pro_2017_02.txt>.
The alignment between GerAB and GkApcT was generated using the HHPred server (https://toolkit.tuebingen.mpg.de/tools/hhpred), and a homology model from the alignment was constructed in MODELLER (https://salilab.org/modeller/) using the structure of GkApcT as a template (PDB ID: 5OQT). Source data are provided with this paper.

Field-specific reporting

Please select the one below that is the best fit for your research. If you are not sure, read the appropriate sections before making your selection.

☒ Life sciences ☐ Behavioural & social sciences ☐ Ecological, evolutionary & environmental sciences

For a reference copy of the document with all sections, see nature.com/documents/nr-reporting-summary-flat.pdf

Life sciences study design

All studies must disclose on these points even when the disclosure is negative.

Sample size	We used more than 1-10 million spores per experiment. This is the standard in the field and far exceeds an amount that could cause error due to under-sampling. All experiments were performed with two technical replicates and with 2 or 3 biological replicates. The variation was minimal. This is a robust assay.
Data exclusions	No data was excluded from our analysis.
Replication	All attempts at replicating our findings were successful. The results reported are robust. All data in the main manuscript and supplementary information were derived from 3 biological replicates with the exception of the germination assays in the supplementary information. These germination assays were derived from two biological replicates each with two technical replicates.
Randomization	No randomization was used. All mutants analyzed were isogenic with matched wild-type controls. All defects in germination or alteration in nutrient specificity were robust.
Blinding	Blinding is not relevant when analyzing bacteria or bacterial spores. Biological replicates with embedded technical replicates ensured reproducibility.

Reporting for specific materials, systems and methods

We require information from authors about some types of materials, experimental systems and methods used in many studies. Here, indicate whether each material, system or method listed is relevant to your study. If you are not sure if a list item applies to your research, read the appropriate section before selecting a response.

Materials & experimental systems

n/a	Involved in the study	
☐	☒	Antibodies
☒	☐	Eukaryotic cell lines
☒	☐	Palaeontology and archaeology
☒	☐	Animals and other organisms
☒	☐	Human research participants
☒	☐	Clinical data
☒	☐	Dual use research of concern

Methods

n/a	Involved in the study	
☒	☐	ChIP-seq
☒	☐	Flow cytometry
☒	☐	MRI-based neuroimaging

Antibodies

Antibodies used	anti-GerAA (a gift from Peter Setlow, Ramirez-Peralta et al., 2012), anti-GFP (Lab Stock, Rudner & Losick 2001), anti-EzrA (a gift from Petra Levin, Haeusser et al., 2004), anti-ScpB (lab stock, Wang et al., 2014), anti-SpoIVFA (a gift from Rich Losick, Resnekov et al., 1996), anti-SleB (a gift from David Popham, Bernhards & Popham, 2014), anti-SigA (a gift from Masaya Fujita, Fujita & Sadaie, 1998), anti-SpoVAD (a gift from Peter Setlow, Vepachedu & Setlow 2005), anti-His (Genscript, catalog number A00186-100), anti-Rabbit (Biorad catalog number 1706515) and anti-Mouse (Biorad catalog number 1706516) secondary antibodies coupled to horseradish peroxidase.<br>With the exception of the anti-His antibody, all primary antibodies were generated by our lab or others and are available upon request. There are no catalog numbers, clone names, or lot numbers to report. We provide this information for the anti-His antibody
Validation	All antibodies were previously validated for specificity in control immunoblots in which lysates from wild-type and a null mutant (or an untagged mutant) were compared. In the few cases in which antibodies were used to analyze an essential protein, we compared lysates from wild-type to a strain harboring a functional YFP fusion to the protein of interest. In this case, we detected a change in

size compared to wild-type.
We describe in detail how all primary antibodies were validated. There are no relevant citations
