# Supplementary material for: Dormant spores sense amino acids through the B subunits of their germination receptors
Source: Nat Commun. 2021 Nov 25;12:6842. doi: 10.1038/s41467-021-27235-2 (PMC8617281; doi:10.1038/s41467-021-27235-2)

Dormant spores sense amino acids through the B subunits of their germination receptors

Lior Artzi^1^, Assaf Alon^2^, Kelly P. Brock^3^, Anna G. Green^3^, Amy Tam^3^, Fernando H. Ramírez-Guadiana^1^, Debora Marks^3^, Andrew Kruse^2^, David Z. Rudner^1^*

^1^ Department of Microbiology, Harvard Medical School, 77 Avenue Louis Pasteur Boston MA 02115

^2^ Department of Biological Chemistry and Molecular Pharmacology, Harvard Medical School, 250 Longwood Avenue Boston MA 02115

^3^ Department of Systems Biology, Harvard Medical School, 200 Longwood Avenue Boston MA 02115

**Original files for Figure 2c**


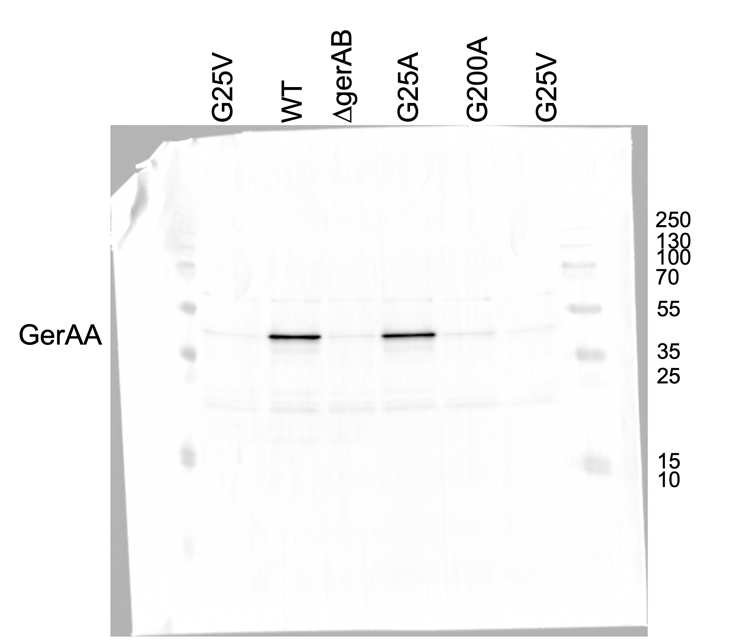


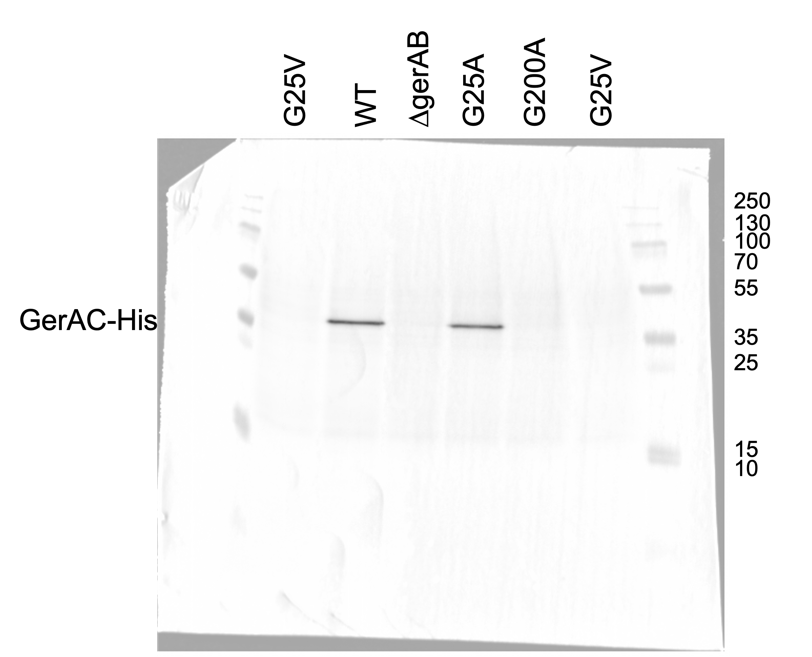


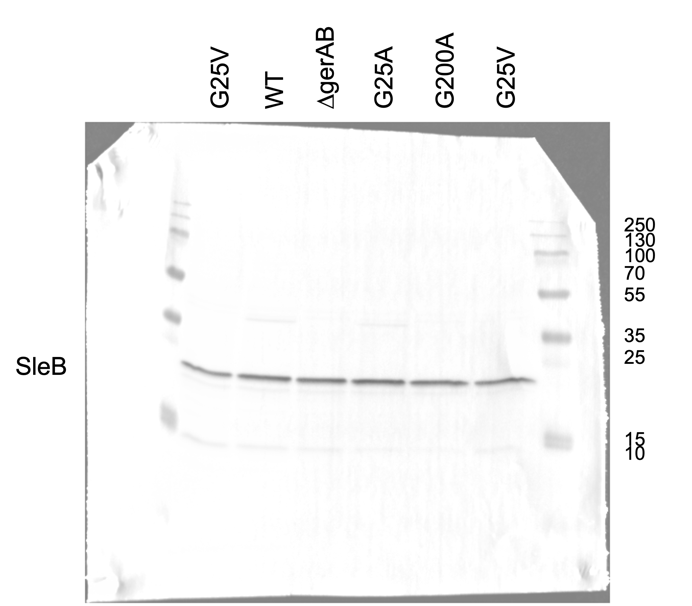


**Original files for Supplementary Figure 3c**


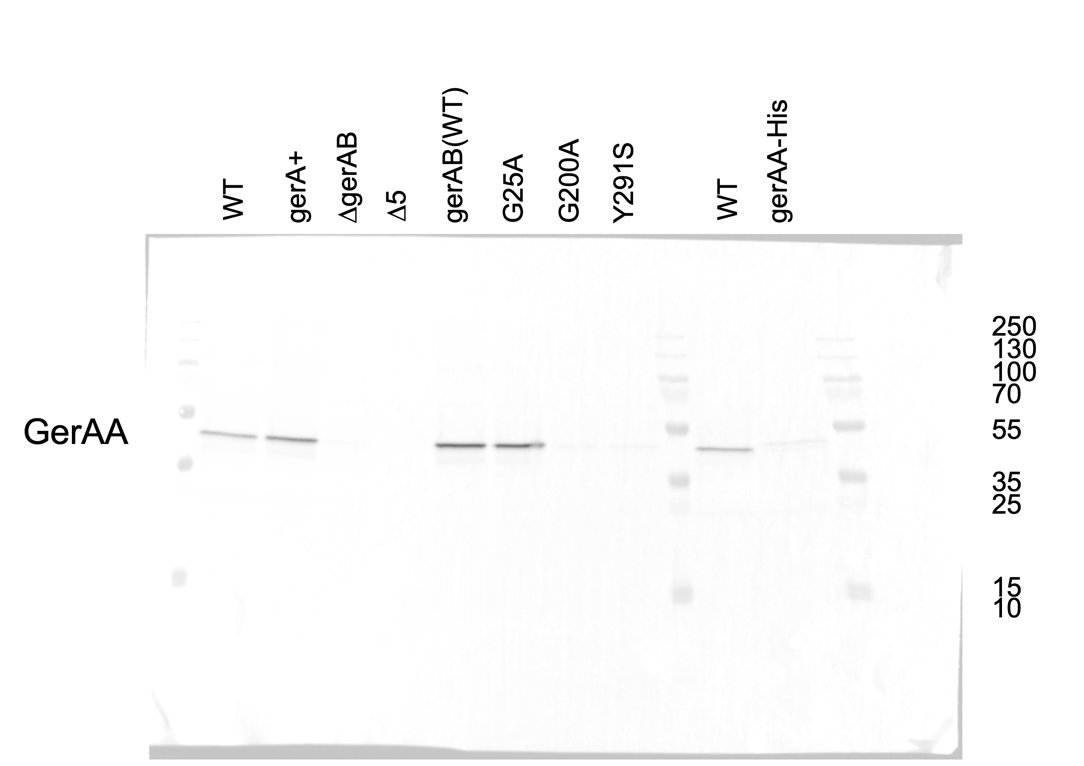


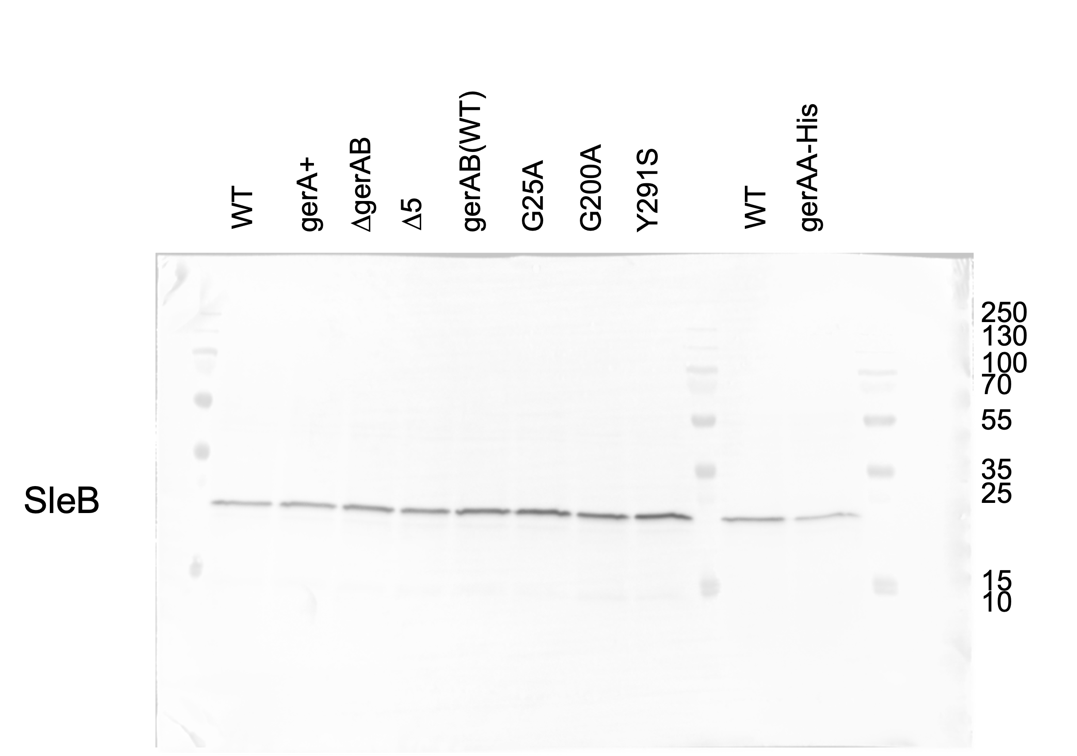


**Original files for Supplementary Figure 7**


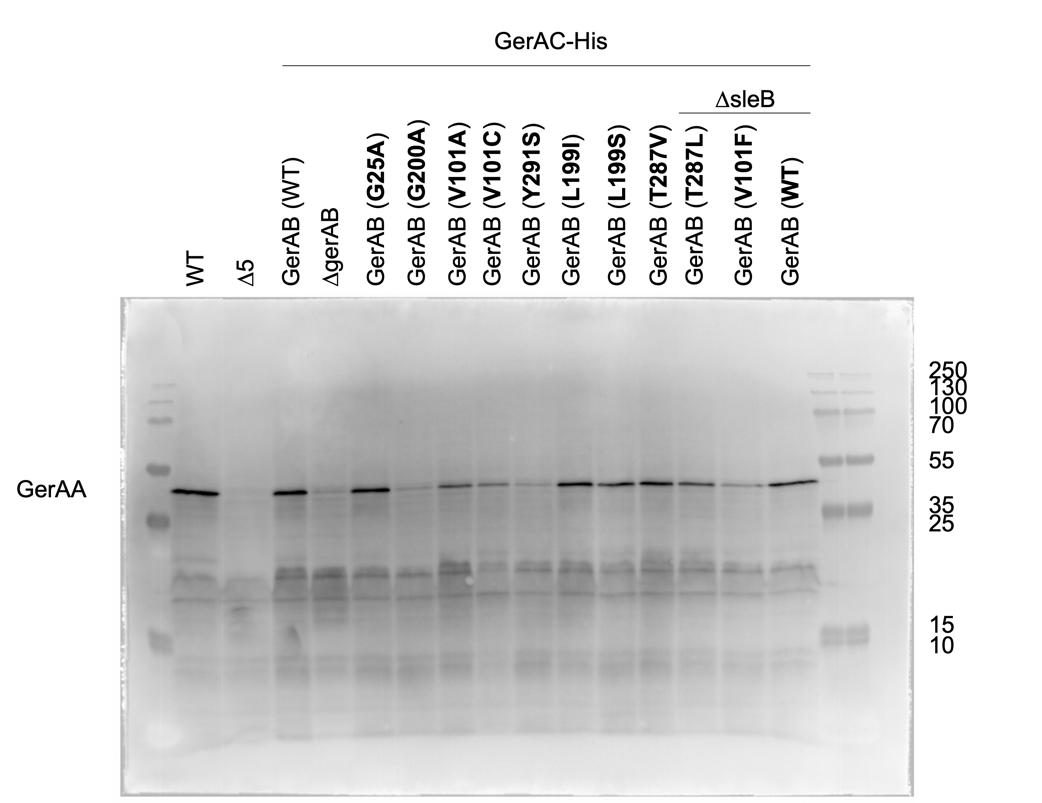


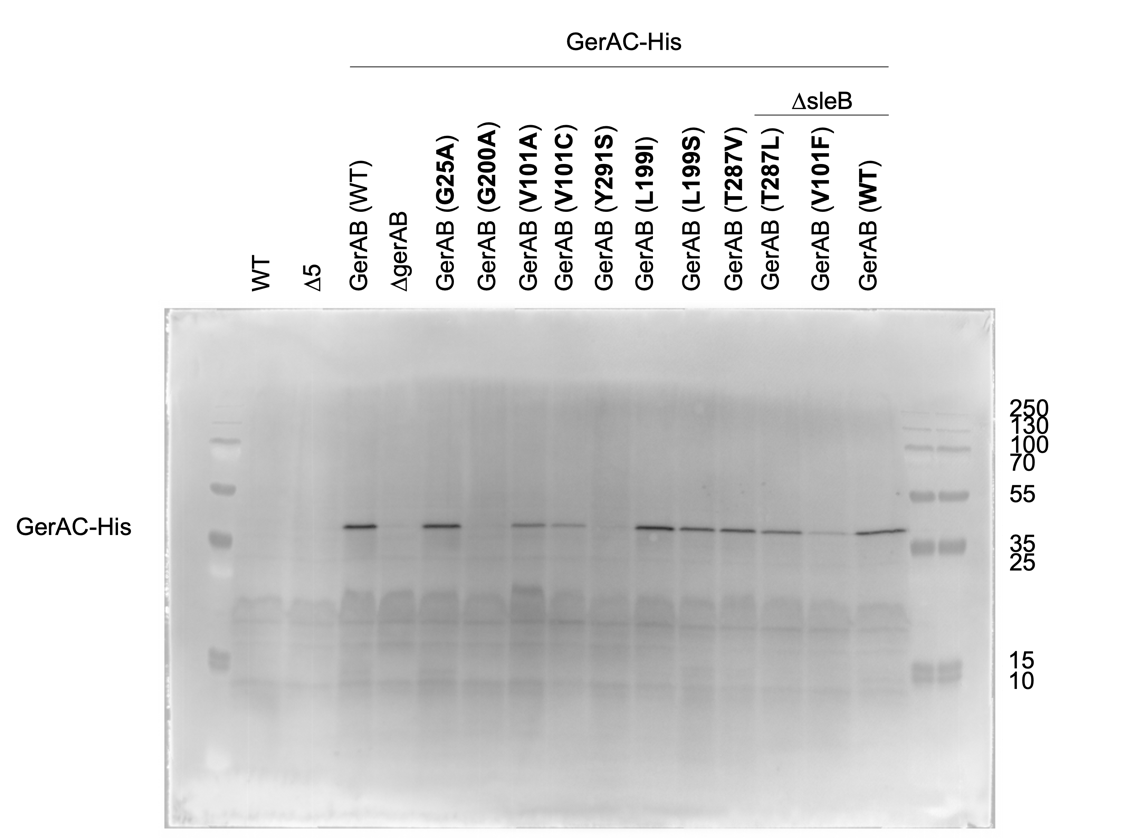


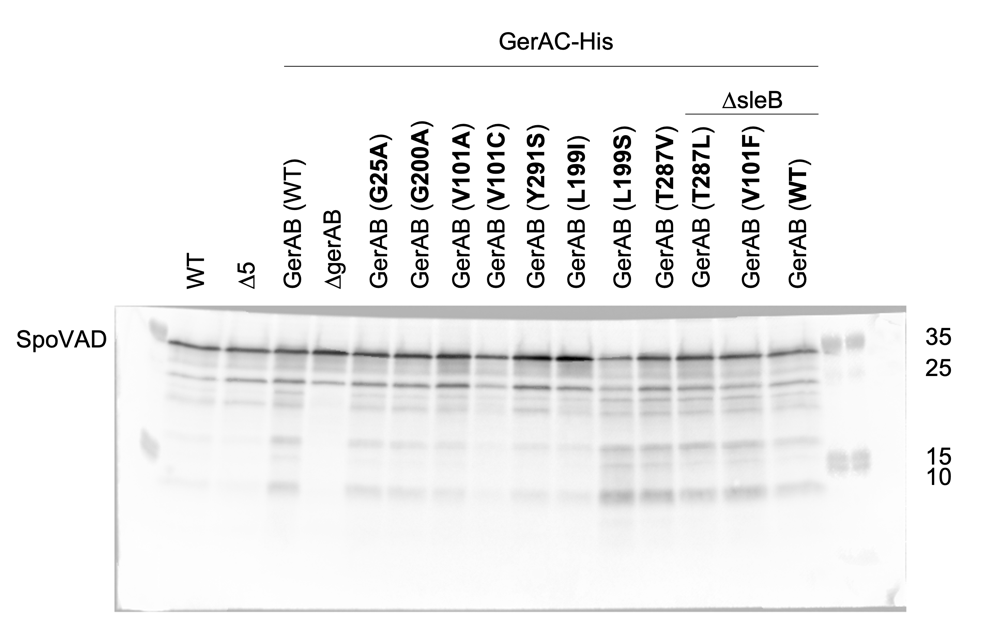


**Original files for Supplementary Figure 12c**


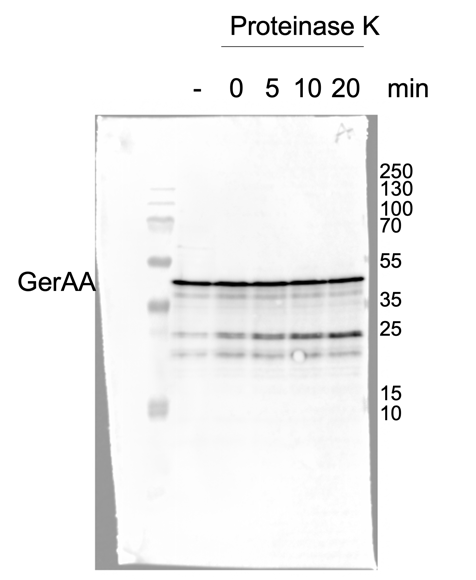


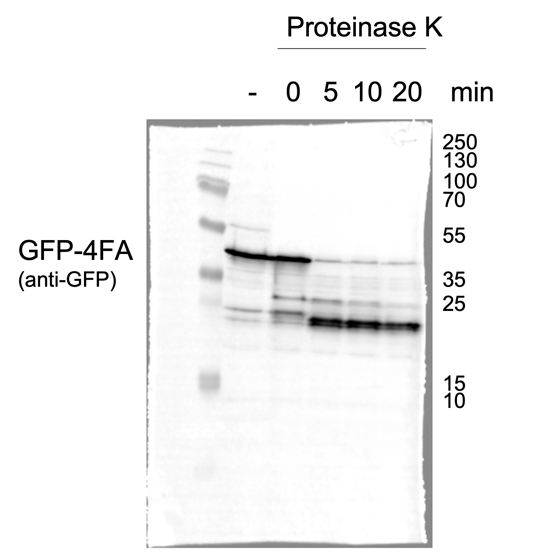


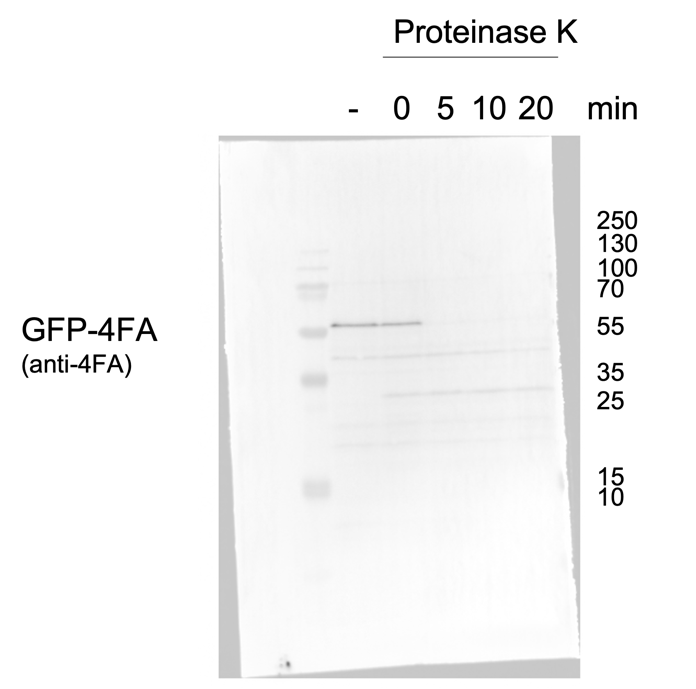


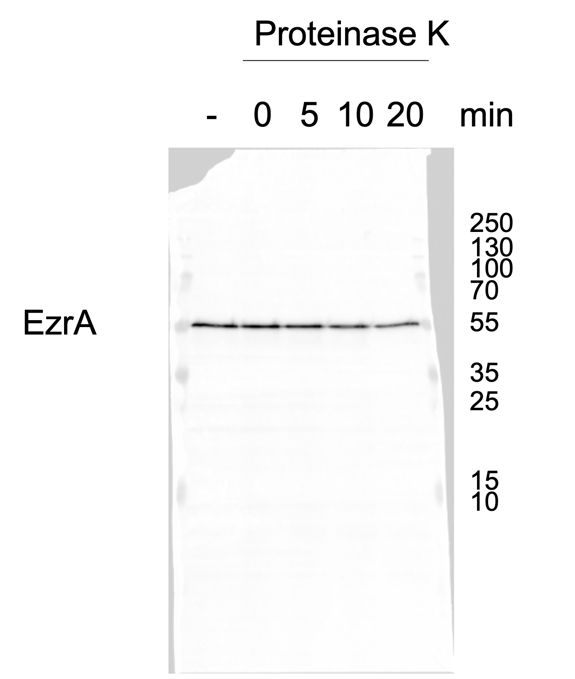


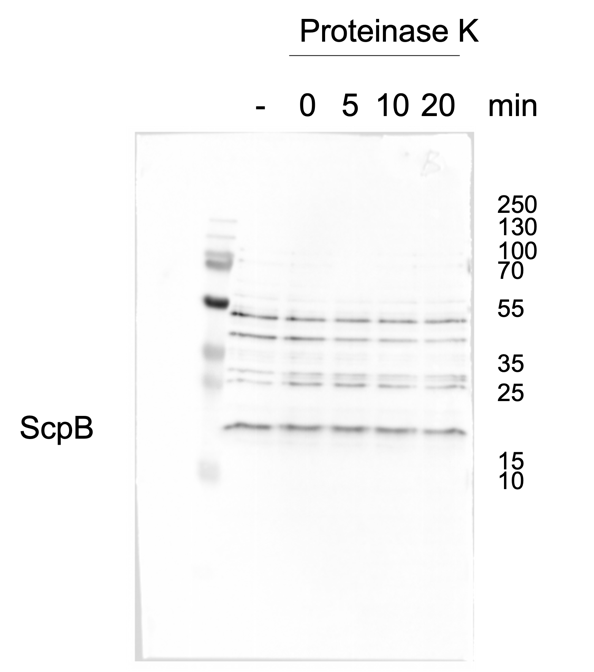


**Original files for Supplementary Figure 12d**


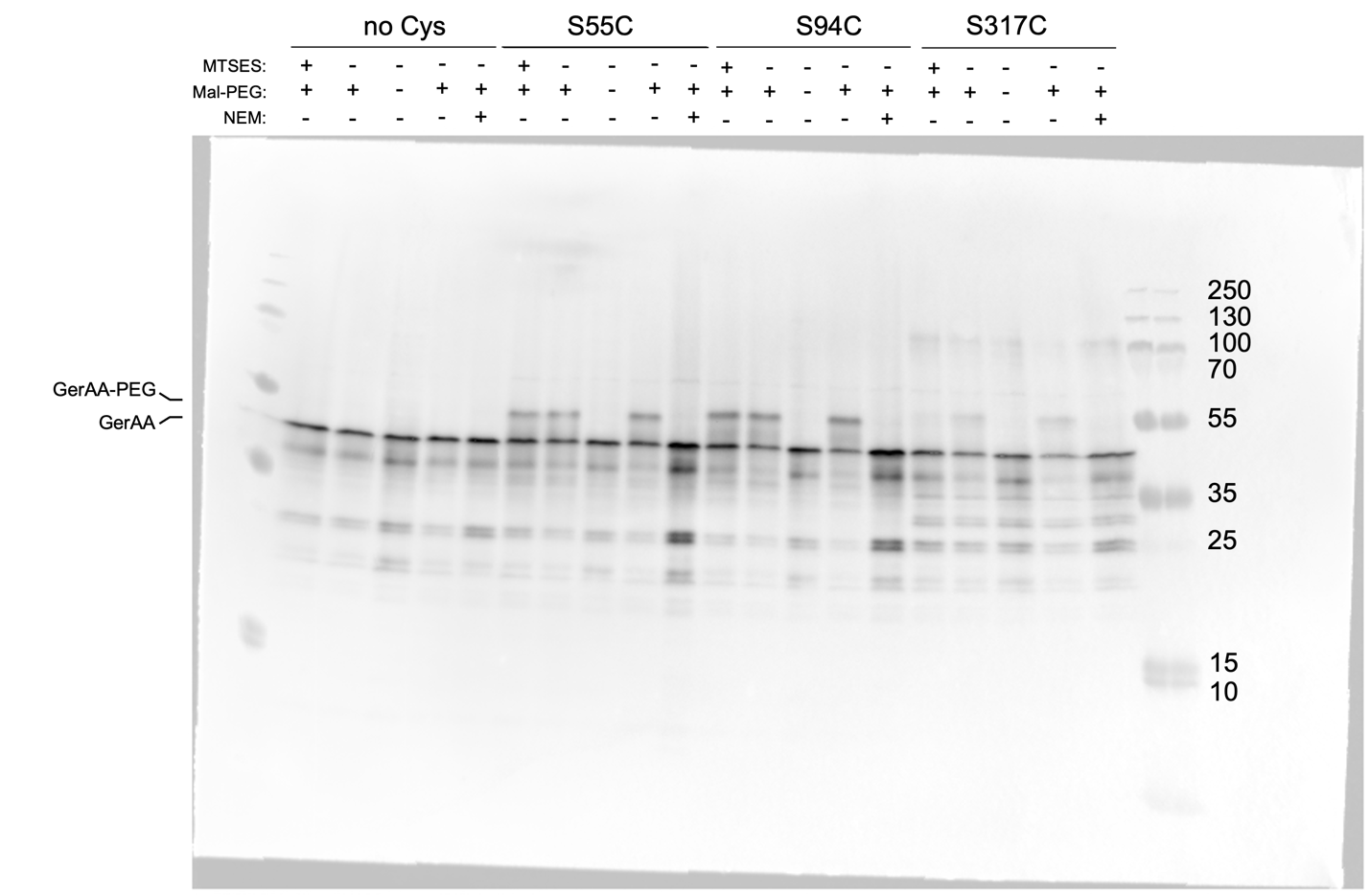


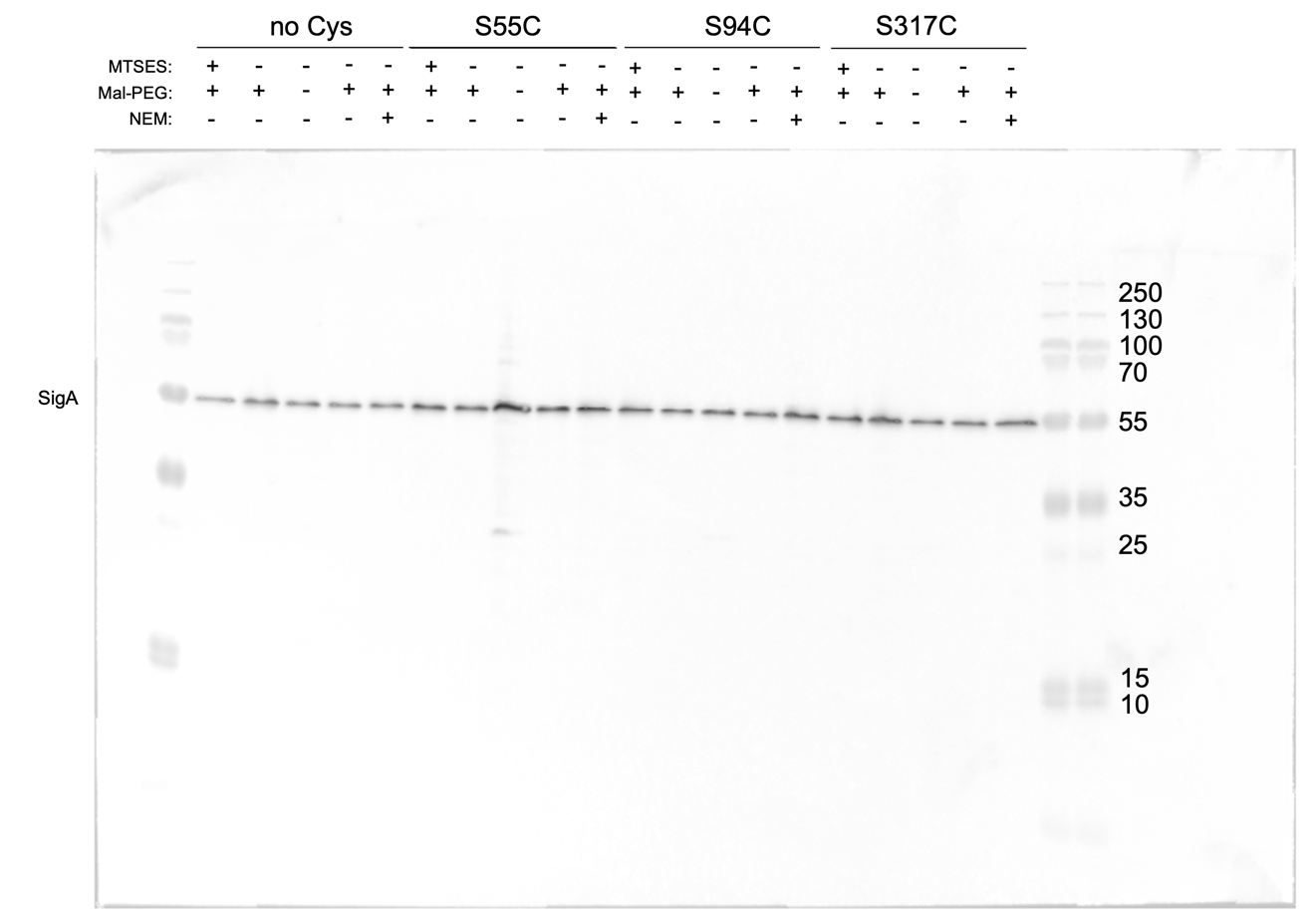

Supplement: Supplementary file 4 — Source Data [file 41467_2021_27235_MOESM4_ESM.zip › Source data/Source Data Artzi et al.docx]
